# A Long Neglected World Malaria Map: *Plasmodium vivax* Endemicity in 2010

**DOI:** 10.1371/journal.pntd.0001814

**Published:** 2012-09-06

**Authors:** Peter W. Gething, Iqbal R. F. Elyazar, Catherine L. Moyes, David L. Smith, Katherine E. Battle, Carlos A. Guerra, Anand P. Patil, Andrew J. Tatem, Rosalind E. Howes, Monica F. Myers, Dylan B. George, Peter Horby, Heiman F. L. Wertheim, Ric N. Price, Ivo Müeller, J. Kevin Baird, Simon I. Hay

**Affiliations:** 1 Spatial Ecology and Epidemiology Group, Department of Zoology, University of Oxford, Oxford, United Kingdom; 2 Eijkman-Oxford Clinical Research Unit, Jakarta, Indonesia; 3 Johns Hopkins Malaria Research Institute, Johns Hopkins Bloomberg School of Public Health, Baltimore, Maryland, United States of America; 4 Fogarty International Center, National Institutes of Health, Bethesda, Maryland, United States of America; 5 Department of Geography and Emerging Pathogens Institute, University of Florida, Gainesville, Florida, United States of America; 6 Oxford University Clinical Research Unit - Wellcome Trust Major Overseas Programme, Ho Chi Minh City, Vietnam; 7 Nuffield Department of Medicine, Centre for Tropical Medicine, University of Oxford, Oxford, United Kingdom; 8 Global Health Division, Menzies School of Health Research, Charles Darwin University, Darwin, Northern Territory, Australia; 9 Division of Medicine, Royal Darwin Hospital, Darwin, Northern Territory, Australia; 10 Papua New Guinea Institute of Medical Research, Goroka, Papua New Guinea; New York University, United States of America

## Abstract

**Background:**

Current understanding of the spatial epidemiology and geographical distribution of *Plasmodium vivax* is far less developed than that for *P. falciparum*, representing a barrier to rational strategies for control and elimination. Here we present the first systematic effort to map the global endemicity of this hitherto neglected parasite.

**Methodology and Findings:**

We first updated to the year 2010 our earlier estimate of the geographical limits of *P. vivax* transmission. Within areas of stable transmission, an assembly of 9,970 geopositioned *P. vivax* parasite rate (*Pv*PR) surveys collected from 1985 to 2010 were used with a spatiotemporal Bayesian model-based geostatistical approach to estimate endemicity age-standardised to the 1–99 year age range (*Pv*PR_1–99_) within every 5×5 km resolution grid square. The model incorporated data on Duffy negative phenotype frequency to suppress endemicity predictions, particularly in Africa. Endemicity was predicted within a relatively narrow range throughout the endemic world, with the point estimate rarely exceeding 7% *Pv*PR_1–99_. The Americas contributed 22% of the global area at risk of *P. vivax* transmission, but high endemic areas were generally sparsely populated and the region contributed only 6% of the 2.5 billion people at risk (PAR) globally. In Africa, Duffy negativity meant stable transmission was constrained to Madagascar and parts of the Horn, contributing 3.5% of global PAR. Central Asia was home to 82% of global PAR with important high endemic areas coinciding with dense populations particularly in India and Myanmar. South East Asia contained areas of the highest endemicity in Indonesia and Papua New Guinea and contributed 9% of global PAR.

**Conclusions and Significance:**

This detailed depiction of spatially varying endemicity is intended to contribute to a much-needed paradigm shift towards geographically stratified and evidence-based planning for *P. vivax* control and elimination.

## Author Summary

*Plasmodium vivax* is one of five parasites causing malaria in humans. Whilst it is found across a larger swathe of the globe and potentially affects a larger number of people than its more notorious cousin, *Plasmodium falciparum*, it receives a tiny fraction of the research attention and financing: around 3%. This neglect, coupled with the inherently more complex nature of vivax biology, means important knowledge gaps remain that limit our current ability to control the disease effectively. This patchy knowledge is becoming recognised as a cause for concern, in particular as the global community embraces the challenge of malaria elimination which, by definition, includes *P. vivax* and the other less common Plasmodium species as well as *P. falciparum*. Particularly conspicuous is the absence of an evidence-based map describing the intensity of *P. vivax* endemicity in different parts of the world. Such maps have proved important for other infectious diseases in supporting international policy formulation and regional disease control planning, implementation, and monitoring. In this study we present the first systematic effort to map the global endemicity of *P. vivax*. We assembled nearly 10,000 surveys worldwide in which communities had been tested for the prevalence of *P. vivax* infections. Using a spatial statistical model and additional data on environmental characteristics and Duffy negativity, a blood disorder that protects against *P. vivax*, we estimated the level of infection prevalence in every 5×5 km grid square across areas at risk. The resulting maps provide new insight into the geographical patterns of the disease, highlighting areas of the highest endemicity in South East Asia and small pockets of Amazonia, with very low endemic setting predominating in Africa. This new level of detailed mapping can contribute to a wider shift in our understanding of the spatial epidemiology of this important parasite.

## Introduction

The international agenda shaping malaria control financing, research, and implementation is increasingly defined around the goal of regional elimination [1]–[6]. This ambition ostensibly extends to all human malarias, but whilst recent years have seen a surge in research attention for *Plasmodium falciparum*, the knowledge-base for the other major human malaria, *Plasmodium vivax*, is far less developed in almost every aspect [7]–[11]. During 2006–2009 just 3.1% of expenditures on malaria research and development were committed to *P. vivax* [12]. The notion that control approaches developed primarily for *P. falciparum* in holoendemic Africa can be transferred successfully to *P. vivax* is, however, increasingly acknowledged as inadequate [13]–[17]. Previous eradication campaigns have demonstrated that *P. vivax* frequently remains entrenched long after *P. falciparum* has been eliminated [18]. The prominence of *P. vivax* on the global health agenda has risen further as evidence accumulates of its capacity in some settings to cause severe disease and death [19]–[25], and of the very large numbers of people living at risk [26].

Amongst the many information gaps preventing rational strategies for *P. vivax* control and elimination, the absence of robust geographical assessments of risk has been identified as particularly conspicuous [9], [27]. The endemic level of the disease determines its burden on children, adults, and pregnant women; the likely impact of different control measures; and the relative difficulty of elimination goals. Despite the conspicuous importance of these issues, there has been no systematic global assessment of endemicity. The Malaria Atlas Project was initiated in 2005 with an initial focus on *P. falciparum* that has led to global maps [28]–[30] for this parasite being integrated into policy planning at regional to international levels [4], [31]–[36]. Here we present the outcome of an equivalent project to generate a comprehensive evidence-base on *P. vivax* infections worldwide, and to generate global risk maps for this hitherto neglected disease. We build on earlier work [26] defining the global range of the disease and broad classifications of populations at risk to now assess the levels of endemicity under which these several billion people live. This detailed depiction of geographically varying risk is intended to contribute to a much-needed paradigm shift towards geographically stratified and evidence-based planning for *P. vivax* control and elimination.

Numerous biological and epidemiological characteristics of *P. vivax* present unique challenges to defining and mapping metrics of risk. Unlike *P. falciparum*, infections include a dormant hypnozoite liver stage that can cause clinical relapse episodes [37], [38]. These periodic events manifest as a blood-stage infection clinically indistinguishable from a primary infection and constitute a substantial, but geographically varying, proportion of total patent infection prevalence and disease burden within different populations [37], [39]–[41]. The parasitemia of *P. vivax* typically occurs at much lower densities compared to those of falciparum malaria, and successful detection by any given means of survey is much less likely. Another major driver of the global *P. vivax* landscape is the influence of the Duffy negativity phenotype [42]. This inherited blood condition confers a high degree of protection against *P. vivax* infection and is present at very high frequencies in the majority of African populations, although is rare elsewhere [43]. These factors, amongst others, mean that the methodological framework for mapping *P. vivax* endemicity, and the interpretation of the resulting maps, are distinct from those already established for *P. falciparum* [28], [29]. The effort described here strives to accommodate these important distinctions in developing a global distribution of endemic vivax malaria.

## Methods

**Figure 1:**
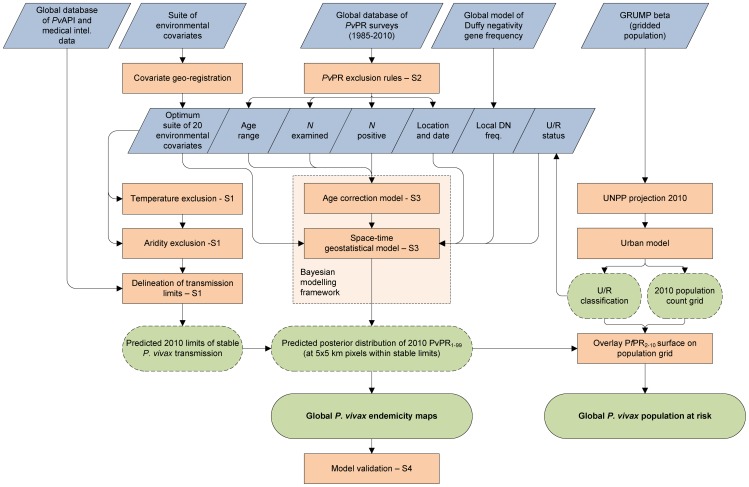
Schematic overview of the mapping procedures and methods for *Plasmodium vivax* endemicity. Blue boxes describe input data. Orange boxes denote models and experimental procedures; green boxes indicate output data (dashed lines represent intermediate outputs and solid lines final outputs). U/R = urban/rural; UNPP = United Nations Population Prospects. Labels S1-4 denote supplementrary information in Protocols S1, S2, S3, and S4.

The modelling framework is displayed schematically in Figure 1. In brief, this involved (i) updating of the geographical limits of stable *P. vivax* transmission based on routine reporting data and biological masks; (ii) assembly of all available *P. vivax* parasite rate data globally; (iii) development of a Bayesian model-based geostatistical model to map *P. vivax* endemicity within the limits of stable transmission; and (iv) a model validation procedure. Details on each of these stages are provided below with more extensive descriptions included as Protocols S1, S2, S3, and S4.

### Updating Estimates of the Geographical Limits of Endemic *Plasmodium vivax* in 2010

The first effort to systematically estimate the global extent of *P. vivax* transmission and define populations at risk was completed in 2009 [26]. As a first step in the current study, we have updated this work with a new round of data collection for the year 2010. The updated data assemblies and methods are described in full in Protocol S1. In brief, this work first involved the identification of 95 countries as endemic for *P. vivax* in 2010. From these, *P. vivax* annual parasite incidence (*Pv*API) routine case reports were assembled from 17,893 administrative units [44]. These *Pv*API and other medical intelligence data were combined with remote sensing surfaces and biological models [45] that identified areas where extreme aridity or temperature regimes would limit or preclude transmission (see Protocol S1). These components were combined to classify the world into areas likely to experience zero, unstable (*Pv*API <0.1‰ per annum), or stable (*Pv*API ≥0.1‰ per annum) *P. vivax* transmission. Despite the very high population frequencies of Duffy negativity across much of Africa, the presence of autochthonous transmission of *P. vivax* has been confirmed by a systematic literature review for 42 African countries [26]. We therefore treated Africa in the same way as elsewhere in this initial stage: regions were deemed to have stable *P. vivax* transmission unless the biological mask layers or *Pv*API data suggested otherwise.

### Creating a Database of Georeferenced *Pv*PR Data

As with *P. falciparum*, the most globally ubiquitous and consistently measured metric of *P. vivax* endemicity is the parasite rate (*Pv*PR), defined as the proportion of randomly sampled individuals in a surveyed population with patent parasitemia in their peripheral blood as detected *via*, generally, microscopy or rapid diagnostic test (RDT). Whilst RDTs can provide lower sensitivity and specificity than conventional blood smear microscopy, and neither technique provides accuracy comparable to molecular diagnostics (such as polymerase chain reaction, PCR), the inclusion of both microscopically and RDT confirmed parasite rate data was considered important to maximise data availability and coverage across the endemic world.

**Figure 2:**
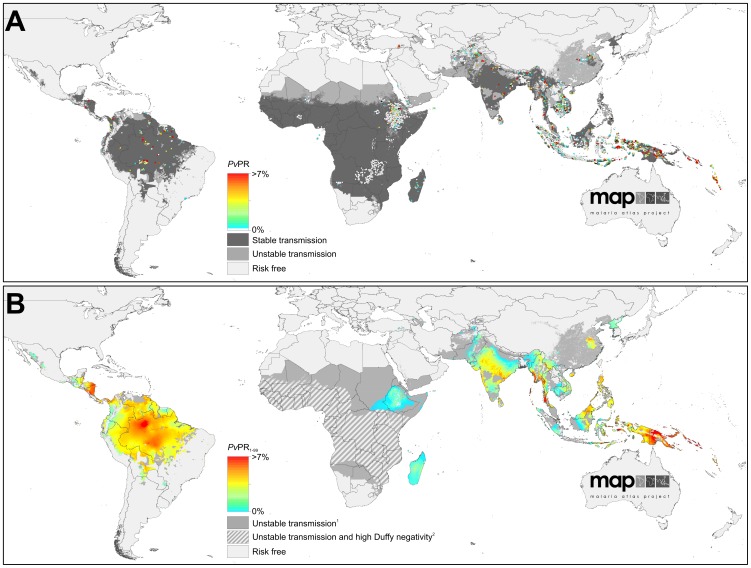
The spatial distribution of *Plasmodium vivax* malaria endemicity in 2010. Panel A shows the 2010 spatial limits of *P. vivax* malaria risk defined by *Pv*API with further medical intelligence, temperature and aridity masks. Areas were defined as stable (dark grey areas, where *Pv*API ≥0.1 per 1,000 pa), unstable (medium grey areas, where *Pv*API <0.1 per 1,000 pa) or no risk (light grey, where *Pv*API = 0 per 1,000 pa). The community surveys of *P. vivax* prevalence conducted between January 1985 and June 2010 are plotted. The survey data are presented as a continuum of light green to red (see map legend), with zero-valued surveys shown in white. Panel B shows the MBG point estimates of the annual mean *Pv*PR_1–99_ for 2010 within the spatial limits of stable *P. vivax* malaria transmission, displayed on the same colour scale. Areas within the stable limits in (A) that were predicted with high certainty (>0.9) to have a *Pv*PR_1–99_ less than 1% were classed as unstable. Areas in which Duffy negativity gene frequency is predicted to exceed 90% [43] are shown in hatching for additional context.

To map endemicity within the boundaries of stable transmission, we first carried out an exhaustive search and assembly of georeferenced *Pv*PR survey data from formal and informal literature sources and direct communications with data generating organisations [46]. Full details of the data search strategy, abstraction and inclusion criteria, geopositioning and fidelity checking procedure are included in Protocol S2. The final database, completed on 25^th^ November 2011, consisted of 9,970 quality-checked and spatiotemporally unique data points, spanning the period 1985–2010. Figure 2A maps the spatial distribution of these data and further summaries by survey origin, georeferencing source, time period, age group, sample size, and type of diagnostic used are provided in Protocol S2.

### Modelling *Plasmodium vivax* Endemicity within Regions of Stable Transmission

We adopt model-based geostatistics (MBG) [47], [48] as a robust and flexible modelling framework for generating continuous surfaces of malaria endemicity based on retrospectively assembled parasite rate survey data [28], [29], [49]. MBG models are a special class of generalised linear mixed models, with endemicity values at each target pixel predicted as a function of a geographically-varying mean and a weighted average of proximal data points. The mean can be defined as a multivariate function of environmental correlates of disease risk. A covariance function is used to characterise the spatial or space-time heterogeneity in the observed data, which in turn is used to define appropriate weights assigned to each data point when predicting at each pixel. This framework allows the uncertainty in predicted endemicity values to vary between pixels, depending on the observed variation, density and sample size of surveys in different locations and the predictive utility of the covariate suite. Parts of the map where survey data are dense, recent, and relatively homogenous will be predicted with least uncertainty, whilst regions with sparse or mainly old surveys, or where measured parasite rates are extremely variable, will have greater uncertainty. When MBG models are fitted using Bayesian inference and a Markov chain Monte Carlo (MCMC) algorithm, uncertainty in the final predictions as well as all model parameters can be represented in the form of predictive posterior distributions [50].

We developed for this study a modified version of the MBG framework used previously to model *P. falciparum* endemicity [28], [29], with some core aspects of the model structure remaining unchanged and others altered to capture unique aspects of *P. vivax* biology and epidemiology. The model is presented in full in Protocol S3. As in earlier work [28], [29], [49], we adopt a space-time approach to allow surveys from a wide time period to inform predictions of contemporary risk. This includes the use of a spatiotemporal covariance function which is parameterised to downweight older data appropriately. We also retain a seasonal component in the covariance function, although we note that seasonality in transmission is often only weakly represented in *Pv*PR in part because of the confounding effect of relapses occurring outside peak transmission seasons [51]. A minimal set of covariates were included to inform prediction of the mean function, based on *a priori* expectations of the major environmental factors modulating endemicity. These were (i) an indicator variable defining areas as urban or rural based on the Global Rural Urban Mapping Project (GRUMP) urban extent product [52], [53]; (ii) a long-term average vegetation index product as an indicator of overall moisture availability for vector oviposition and survival [54], [55]; and (iii) a *P. vivax* specific index of temperature suitability derived from the same model used to delineate suitable areas on the basis of vector survival and sporogony [45].

### Age Standardisation

Our assembly of *Pv*PR surveys was collected across a variety of age ranges and, since *P. vivax* infection status can vary systematically in different age groups within a defined community, it was necessary to standardise for this source of variability to allow all surveys to be used in the same model. We adopted the same model form as has been described [56] and used previously for *P. falciparum* [28], [29], whereby population infection prevalence is expected to rise rapidly in early infancy and plateau during childhood before declining in early adolescence and adulthood. The timing and relative magnitude of these age profile features are likely distinct between the two parasites in different endemic settings [51], [57], and so the model was parameterised using an assembly of 67 finely age-stratified *Pv*PR surveys (Protocol S2), with estimation carried out in a Bayesian model using MCMC. The parameterised model was then used to convert all observed survey prevalences to a standardised age-independent value for use in modelling, and then further allowed the output prevalence predictions to be generated for any arbitrary age range. We chose to generate maps of all-age infection prevalence, defined as individuals of age one to 99 years (thus *Pv*PR_1–99_). We excluded infection in those less than one year of age from the standardisation because of the confounding effect of maternal antibodies, and because parasite rate surveys very rarely sample young infants. We deviated from the two-to-ten age range used for mapping *P. falciparum* [28], [29] because the relatively lower prevalences has meant that surveys are far more commonly carried out across all age ranges.

### Incorporating Duffy Negativity

Since Duffy negative individuals are largely refractory to *P. vivax* infection [58], high population frequencies of this phenotype have a dramatic suppressing effect on endemicity, even where conditions are otherwise well suited for transmission [26]. The predominance of Duffy negativity in Africa has led to a historical perception that *P. vivax* is absent from much of the continent, and a dearth of surveys or routine diagnoses testing for the parasite have served to entrench this mantra [59]. However, evidence exists of autochthonous *P. vivax* transmission across the continent [26], and therefore we did not preclude any areas at risk *a priori*. Instead, we used a recent map of estimated Duffy negativity phenotypic frequency [43] and incorporated the potential influence of this blood group directly in the MBG modelling framework. The mapped Duffy-negative population fraction at each location was excluded from the denominator in *Pv*PR survey data, such that any *P. vivax* positive individuals were considered to have arisen from the Duffy positive population subset. Thus in a location with 90% Duffy negativity, five positive individuals in a survey of 100 would give an assumed prevalence of 50% amongst Duffy positives. Correspondingly, prediction of *Pv*PR was then restricted to the Duffy positive proportion at each pixel, with the final prevalence estimate re-converted to relate to the total population. This approach has two key advantages. First, predicted *Pv*PR at each location could never exceed the Duffy positive proportion, therefore ensuring biological consistency between the *P. vivax* and Duffy negativity maps. Second, where *Pv*PR survey data were sparse across much of Africa, the predictions could effectively borrow strength from the Duffy negativity map because predictions of *Pv*PR were restricted to a much narrower range of possible values.

### Model Implementation and Map Generation

The *P. vivax* endemic world was divided into four contiguous regions with broadly distinct biogeographical, entomological and epidemiological characteristics: the Americas and Africa formed separate regions, whilst Asia was subdivided into Central and South East sub-regions with a boundary at the Thailand-Malaysia border (see Protocol S2). This regionalisation was implemented in part to retain computational feasibility given the large number of data points, but also to allow model parameterisations to vary and better capture regional endemicity characteristics. Within each region, a separate MBG model was fitted using a bespoke MCMC algorithm [60] to generate predictions of *Pv*PR_1–99_ for every 5×5 km pixel within the limits of stable transmission. The prediction year was set to 2010 and model outputs represent an annualised average across the 12 months of that year. Model output consisted of a predicted posterior distribution of *Pv*PR_1–99_ for every pixel. A continuous endemicity map was generated using the mean of each posterior distribution as a point estimate. The uncertainty associated with predictions was summarised by maps showing the ratio of the posterior distribution inter-quartile range (IQR) to its mean. The IQR is a simple measure of the precision with which each *Pv*PR value was predicted, and standardisation by the mean produced an uncertainty index less affected by underlying prevalence levels and more illustrative of relative model performance driven by data densities in different locations. This index was then also weighted by the underlying population density to produce a second map indicative of those areas where uncertainty is likely to be most operationally important.

### Refining Limits Definition and Population at Risk Estimates

In some regions within the estimated limits of stable transmission, *Pv*PR_1–99_ was predicted to be extremely low, either because of a dense abundance of survey data reporting zero infections or, in Africa, because of very high coincident Duffy negativity phenotype frequencies. Such areas are not appropriately described as being at risk of stable transmission and so we defined a decision rule whereby pixels predicted with high certainty (probability >0.9) of being less than 1% *Pv*PR_1–99_ were assigned to the unstable class, thereby modifying the original transmission limits. These augmented mapped limits were combined with a 2010 population surface derived from the GRUMP *beta* version [52], [53] to estimate the number of people living at unstable or stable risk within each country and region. The fraction of the population estimated to be Duffy negative [43] within each pixel was considered at no risk and therefore excluded from these totals.

### Model Validation

A model validation procedure was implemented whereby 10% of the survey points in each model region were selected using a spatially declustered random sampling procedure. These subsets were held out and the model re-fitted in full using the remaining 90%. Model predictions were then compared to the hold-out data points and a number of different aspects of model performance were assessed using validation statistics described previously [28], [29]. The validation procedure is detailed in full in Protocol S4.

## Results

### Model Validation

Full validation results are presented in Protocol S4. In brief, examination of the mean error in the generation of the *P. vivax* malaria endemicity point-estimate surface revealed minimal overall bias in predicted *Pv*PR with a global mean error of −0.41 (Americas −1.38, Africa 0.03, Central Asia −0.43, South East Asia −0.43), with values in units of *Pv*PR on a percentage scale (see Protocol S4). The global value thus represents an overall tendency to underestimate prevalence by just under half of one percent. The mean absolute error, which measures the average magnitude of prediction errors, was 2.48 (Americas 5.05, Africa 0.53, Central Asia 1.52, South East Asia 3.37), again in units of *Pv*PR (see Protocol S4).

### Global *Plasmodium vivax* Endemicity and Populations at Risk in 2010

**Figure 3:**
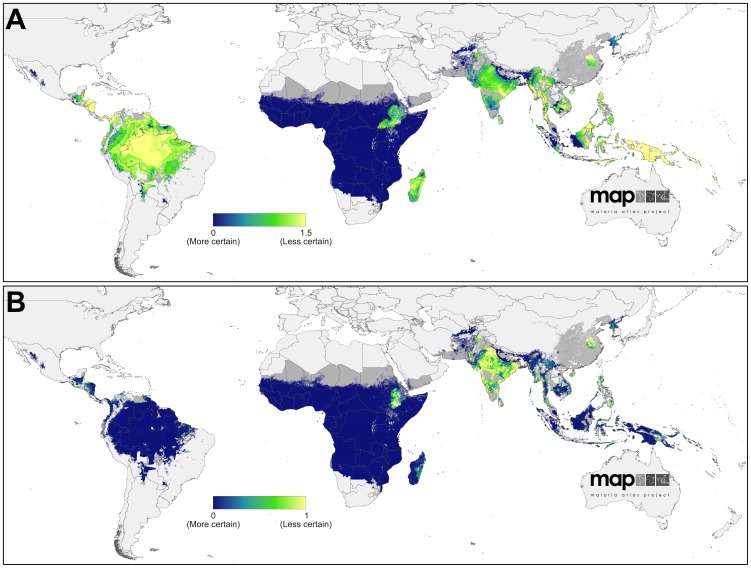
Uncertainty associated with predictions of *Plasmodium vivax* endemicity. Panel A shows the ratio of the posterior inter-quartile range to the posterior mean prediction at each pixel. Large values indicate greater uncertainty: the model predicts a relatively wide range of *Pv*PR_1–99_ as being equally plausible given the surrounding data. Conversely, smaller values indicate a tighter range of values have been predicted and, thus, a higher degree of certainty in the prediction. Panel B shows the same index multiplied by the underlying population density and rescaled to 0–1 to correspond to Panel A. Higher values indicate areas with high uncertainty and large populations.

The limits of stable and unstable *P. vivax* transmission, as defined using *Pv*API, biological exclusion masks and medical intelligence data are shown in Figure 2A. The continuous surface of *P. vivax* endemicity predicted within those limits is shown in Figure 2B. The uncertainty map (posterior IQR:mean ratio) is shown in Figure 3A and the population-weighted version in Figure 3B.

We estimate that *P. vivax* was endemic across some 44 million square kilometres, approximately a third of the Earth's land surface. Around half of this area was located in Africa (51%) and a quarter each in the Americas (22%) and Asia (27%) (Table 1). However, the uneven distribution of global populations, coupled with the protective influence of Duffy negativity in Africa, meant that the distribution of populations at risk was very different. An estimated 2.48 billion people lived at any risk of *P. vivax* in 2010 (Table 1), of which a large majority lived in Central Asia (82%) with much smaller fractions in South East Asia (9%), the Americas (6%), and Africa (3%). Of these, 1.52 billion lived in areas of unstable transmission where risk is very low and case incidence is unlikely to exceed one per 10,000 per annum. The remaining 964 million people at risk lived in areas of stable transmission, representing a wide diversity of endemic levels. The global distribution of populations in each risk class was similar to the total at risk, such that over 80% of people in both classes lived in Central Asia (Table 1).

**Table 1:** Area and populations at risk of *Plasmodium vivax* malaria in 2010.

Region	Area (million km^2^)			Population (millions)		
	Unstable	Stable	Any risk	Unstable	Stable	Any risk
America	1.38	8.08	9.46	87.66	49.79	137.45
Africa+	20.60	1.86	22.46	48.72	37.66	86.38
C Asia	5.60	3.63	9.24	1,236.92	812.55	2,049.47
SE Asia	0.96	1.78	2.74	150.17	64.90	215.07
World	28.55	15.35	43.90	1,523.47	964.90	2,488.37

Risk is stratified into unstable risk (*Pv*API<0.1 per 1,000 people pa) and stable risk (*Pv*API≥0.1 per 1,000 people pa).

### *Plasmodium vivax* Endemicity in the Americas

Areas endemic for *P. vivax* in the Americas extended to some 9.5 million square kilometres, of which the largest proportion was in the Amazonian region of Brazil (Figure 2B). Interestingly, only a relatively small fraction of these areas (15%) experienced unstable rather than stable transmission, suggesting a polarisation between areas at stable risk and those where the disease is absent altogether (Table 1). The regions of highest endemicity were found in Amazonia and in Central America – primarily Nicaragua and Honduras – with predicted mean *Pv*PR_1–99_ exceeding 7% in all three locations. An important feature of *P. vivax* throughout the Americas is that its distribution is approximately inverse to that of the population. This is particularly true of the two most populous endemic countries of the region, Brazil and Mexico, and it means that, whilst the Americas contributed 53% of the land area experiencing stable transmission worldwide, they housed only 5% of the global population at that level of risk.

Uncertainty in predicted *Pv*PR_1–99_ was relatively high throughout much of the Americas (Figure 3B). This reflects the heterogeneous landscape of endemicity coupled with the generally scarce availability of parasite rate surveys in the region (see Figure 2A). However, when this uncertainty is weighted by the underlying population density (Figure 3B), its significance on a global scale is placed in context: because most areas at stable risk are sparsely populated, the population-weighted uncertainty was very low compared to parts of Africa and much of Asia.

### *Plasmodium vivax* Endemicity in Africa, Yemen and Saudi Arabia (Africa+)

Our decision to assume stable transmission of *P. vivax* in Africa unless robust *Pv*API or biological mask data confirmed otherwise meant that much of the continent south of the Sahara was initially classified as being at stable risk (Figure 2A). However, by implementing the MBG predictions of *Pv*PR_1–99_ throughout this range and reclassifying *a posteriori* those areas likely to fall below an endemicity threshold of 1% *Pv*PR_1–99_, the majority of stable risk areas were downgraded to unstable (Figure 2B). Thus, in the final maps, 92% of endemic Africa was at unstable risk, with the majority of Madagascar and Ethiopia, and parts of South Sudan and Somalia making up most of the remaining area at stable risk. Even in these areas, endemicity was uniformly low, with predicted endemicity values rarely exceeding a point estimate of 2% *Pv*PR_1–99_. We augmented the final map with an additional overlay mask delineating areas where Duffy negativity phenotype prevalence has been predicted to exceed 90% (Figure 2B). The influence of this blood group on the estimated populations at risk is profound: of the 840 million Africans living in areas within which transmission is predicted to occur, only 86 million were considered at risk, contributing just 3% to the global total (Table 1).

Uncertainty in predicted *Pv*PR_1–99_ followed a similar pattern to the magnitude of the predictions themselves (Figure 3B). Certainty around the very low predicted endemicity values covering most of the continent was extremely high – reflecting the increased precision gained by incorporating the Duffy negativity information that compensated for the paucity of *P. vivax* parasite rate surveys on the continent. The pockets of higher endemicity in Madagascar and northern East Africa were predicted with far less certainty. In the population-weighted uncertainty map (Figure 3B), the lower population densities of Madagascar reduced the index on that island whereas the densely populated Ethiopian highlands remained high.

### *Plasmodium vivax* Endemicity in Central and South East Asia

Large swathes of high endemicity, very large population densities and a negligible presence of Duffy negativity combine to make the central and south-eastern regions of Asia by far the most globally significant for *P. vivax*. We estimate that India alone contributed nearly half (46%) of the global population at risk, and two thirds (67%) of those at stable risk. China is another major contributor with 19% of the global populations at risk, primarily in unstable transmission regions, whilst Indonesia and Pakistan together contributed a further 12%. Within regions of stable transmission, endemicity is predicted to be extremely heterogeneous (Figure 2B). Areas where the point estimate of *Pv*PR_1–99_ exceeded 7% were found in small pockets of India, Myanmar, Indonesia, and the Solomon Islands, with the largest such region located in Papua New Guinea.

The uncertainty map (Figure 3A) reveals how the most precise predictions were associated with areas of uniformly low endemicity and abundant surveys, such as Afghanistan and parts of Sumatra and Kalimantan in Indonesia. Conversely, areas with higher or more heterogeneous endemicity, such as throughout the island of New Guinea, were the most uncertain. The population-weighted uncertainty map (Figure 3B) differs substantively, indicating how the populous areas of Indonesia, for example, were relatively precisely predicted whereas India, China, and the Philippines had the largest per-capita uncertainty.

## Discussion

The status of *P.* vivax as a major public health threat affecting the world's most populous regions is becoming increasingly well documented. The mantra of vivax malaria being a very rarely threatening and relatively benign disease [7], [10] has been challenged with evidence suggesting that it can contribute a significant proportion of severe malaria disease and death attributable to malaria in some settings [61]. Some reports have pointed especially to very young children being a major source of morbidity [20], [62] and some hospital-based studies have reported comparable mortality rates between patients classified with severe *P. vivax* and severe *P. falciparum* [21], [24], [63]. The recognition of a lethal threat by this parasite comes with evidence of failing chemotherapeutics against the acute attack [64] and overdue acknowledgement of the practical inadequacy of the only available therapy against relapse [65]. As the international community defines increasingly ambitious targets to minimise malaria illness and death [66]–[68], and to progressively eliminate the disease from endemic areas [1]–[6], further sustained neglect of *P. vivax* becomes increasingly untenable.

Here we have presented the first systematic attempt to map the global distribution of *P. vivax* endemicity using a defined evidence base, transparent methodologies, and with measured uncertainty. These new maps aim to contribute to a more rational international appraisal of the importance of *P. vivax* in the broad context of malaria control and elimination policies, as well as providing a practical tool to support control planning at national and sub-national levels.

### Interpreting *P. vivax* Endemicity in 2010

In 2010, areas endemic for *P. vivax* covered a huge geographical range spanning three major continental zones and extending into temperate climates. In the Americas, whilst important pockets of high endemicity are present, the majority of areas of stable transmission coincide with lower population densities, diminishing the contribution of this continent to global populations at risk. In Africa the protection conferred by Duffy negativity to most of the population means the large swathes of the continent in which transmission may occur contain only small populations at biological risk. Thus it is primarily in Asia where very large populations coincide with extensive high endemic regions, and as a result nine out of every ten people at risk of *P. vivax* globally live on that continent.

A number of important contrasts arise when comparing this map with the equivalent 2010 iteration for *P. falciparum* [28]. Perhaps most obvious are the lower levels of observed endemicity at which *P. vivax* tends to exist within populations experiencing stable transmission. We used a cartographic scale between 0% and 7% to differentiate global variation in *P. vivax* endemicity, although point estimates exceeded that upper threshold in localised areas. For *P. falciparum* the equivalent scale spanned 0% to 70% [28], suggesting an approximate order-of-magnitude difference in prevalence of patent parasitemia. In part, this difference reflects the decision to standardise our predictions across the 1–99 age range, and values would have been higher if we had opted for the peak 2–10 age range used for *P. falciparum*. This difference might be accentuated by the likely more rapid acquisition of immunity to *P. vivax* than *P. falciparum* in the most highly endemic areas [57]. A number of other biological and epidemiological differences between the two species also mean these lower apparent levels of endemicity must be interpreted differently. One factor is the lower sensitivities of microscopy and RDT diagnoses for a given level of *P. vivax* infection prevalence, because infections tend to be associated with much lower parasite densities which increase the likelihood of false negative diagnoses [9]. A number of studies in both high and low endemic settings have found microscopy to underestimate prevalence by a factor of up to three when compared with molecular diagnosis [57], [69]–[72]. The decreasing cost and time implications of molecular diagnosis may mean that these gold standard diagnostic techniques become the standard for parasite rate surveys in the future. A global map of PCR-positive parasitemia rates would almost certainly reveal a larger underlying reservoir of infections and, possibly, reveal systematic differences in patterns of endemicity than we are able to resolve currently with less sensitive diagnostic methods.

The lower parasite loads must be interpreted in the context of implications for progression to clinical disease. For example, *Plasmodium vivax* is known to induce fevers at comparatively lower parasite densities than *P. falciparum*, a feature likely linked to overall inflammatory responses of greater magnitude [16]. *P. vivax* is also comparable to *P. falciparum* in its potential to cause anaemia regardless of lower parasite densities, due to a combination of dyserythropoesis and repeated bouts of haemolysis [22]. A recent hospital-based study at a site in eastern Indonesia of hypo- to meso-endemic transmission of both species showed far lower frequencies of parasitemia >6,000/uL among inpatients classified as having not serious, serious, and fatal illness with a diagnosis of *P. vivax* compared to *P. falciparum* [24]. Further, the majority of case reports describing severe and fatal illness with a diagnosis of vivax malaria typically show parasitemia >5,000/uL. In contrast, the World Health Organization threshold for severe illness attributable to hyperparasitemia with *P. falciparum* is >200,000/uL [73]. In brief, the relationship between prevalence and risk of disease and transmission for *P. vivax* is distinct from that for *P. falciparum*, and it is weighted more heavily towards substantial risks at much lower parasite densities and levels of prevalence of microscopically patent parasitemia.

The capacity of *P. vivax* hypnozoites to induce relapsing infections has a number of important implications. First, because dormant liver stage infections are not detectable in routine parasite rate surveys, our maps do not capture the potentially very large reservoir of asymptomatic infections sequestered in each population. Evidence is emerging that this hidden reservoir may be substantially larger than previously thought, with long-latency *P. vivax* phenotypes both prevalent and geographically widespread [37]. Whilst not contributing to clinical disease until activated, these dormant hypnozoites ultimately play a vital role in sustaining transmission since they are refractory to blood-stage antimalarial chemotherapy and interventions to reduce transmission. Hypnozoites also ensure an ability of *P. vivax* to survive in climatic conditions that cannot sustain *P. falciparum* transmission. Second, the *P. vivax* parasite rates observed in population surveys detect both new and relapsing infections, although the two are almost never distinguishable. This confounds the relationship between observed infection prevalence and measures of transmission intensity such as force of infection or the entomological inoculation rate. This, in turn, has implications for the use of transmission models seeking to evaluate or optimise control options for *P. vivax* [2], [9], [27], [74]. The current unavailability of any diagnostic method for detecting hypnozoites [75] and our resulting ignorance about the size and geographic distribution of this reservoir therefore remain critical knowledge gaps limiting the feasibility of regional elimination [9]. It is also worth noting that conventional parasite rate data do not measure multiplicity of infection which is an additional potential confounding effect between observed infection prevalence and transmission intensity.

### *P. vivax* in Africa and Duffy Polymorphism

Our map of *P. vivax* endemicity and estimates of populations at risk in Africa are heavily influenced by a single assumption: that the fraction of the population estimated to be negative for the Duffy antigen [43] is refractory to infection with *P. vivax*. A body of empirical evidence is growing, however that *P. vivax* can infect and cause disease in Duffy negative individuals, as reported in Madagascar [76] and mainland sub-Saharan Africa [77]–[80] as well as outside Africa [81], [82]. Whether the invasion of erythrocytes *via* Duffy antigen-independent pathways is a newly evolved mechanism, or whether this capacity has been overlooked by the misdiagnosis of *P. vivax* in Africa as *P. ovale* remains unresolved [9], [42], [59]. Whilst this accumulated evidence stands contrary to our simplifying assumption of complete protection in Duffy negative individuals, there is currently no evidence to suggest that such infections are anything but rare and thus are unlikely to have any substantive influence on the epidemiology or infection prevalence of *P. vivax* at the population scale throughout most of Africa. We also make no provision in our model for a protective effect in Duffy-negative heterozygotes, although such protection has been observed in some settings [83]–[86]. The movement and mixing within Africa of human populations from diverse ethnographic backgrounds complicates contemporary patterns of Duffy negativity and, in principle, could yield local populations with substantially reduced protection from *P. vivax* infection in the future. Indeed, the implications for our map of population movement go beyond the effect of Duffy negativity: the carriage of parasites from high to low endemic regions, for example by migratory workers, may play an important role in sustaining transmission in some regions and further research is required to investigate such processes.

### Mapping to Guide Control

There exists for *P. falciparum* a history of control strategies linked explicitly to defined strata of endemicity, starting with the first Global Malaria Eradication Programme [18], [87], [88] and undergoing a series of refinements that now feature in contemporary control and elimination efforts. Most recently, stratification has been supported by insights gained from mathematical models linking endemic levels to optimum intervention suites, control options, and timelines for elimination planning [2], [89]–[95]. In stark contrast, control options for *P. vivax* are rarely differentiated by endemicity, and there is little consensus around how this may be done. In part, the absence of agreed control-oriented strata of *P. vivax* endemicity stems from the biological complexities and knowledge gaps that prevent direct interpretation of infection prevalence as a metric for guiding control. It is also to some extent inevitable that the dogma of unstratified control becomes self-propagating: risk maps are not created because control is not differentiated by endemicity, but that differentiation cannot proceed without reliable maps.

As well as providing a basis for stratified control and treatment, the endemicity maps presented here have a number of potential applications in combination with other related maps. First, there is an urgent need to better identify regions where high *P. vivax* endemicity is coincident with significant population prevalence of glucose-6-phosphate dehydrogenase deficiency (G6PDd). This inherited blood disorder plays a key role in chemotherapy policy for *P. vivax* because primaquine, the only registered drug active against the hypnozoite liver stage is contra-indicated in G6PDd individuals in whom it can cause severe and potentially fatal haemolytic reactions [96], [97]. A new global map of G6PDd prevalence is now available (Howes et al, submitted) which can be combined with the endemicity maps presented here to provide a rational basis for estimating adverse outcomes and setting appropriate testing and treatment protocols. Moreover, in practice most clinical infections are managed without differentiating the causative parasite species: combining the endemicity maps for *P. vivax* and *P. falciparum* may therefore inform unified strategies for malaria control programs and policy [28]. It has been proposed, for example, that artemesinin-based combination therapy (ACT) be adopted for all presumptively diagnosed malaria in areas coendemic for both species, as opposed to a separate ACT/chloroquine treatment strategy [98]. Further, in some regions more than 50% of patients diagnosed with falciparum malaria go on to experience an attack of vivax malaria in the absence of risk of reinfection [99]. This high prevalence of hypnozoites may also justify presumptive therapy with primaquine against relapse with any diagnosis of malaria where the two species occur at relatively high frequencies. Such geographically specific cross-parasite treatment considerations hinge on robust risk maps for both species.

### Future Challenges in *P. vivax* Cartography

Numerous research and operational challenges remain unaddressed that would provide vital insights into the geographical distribution of *P. vivax* and its impacts on populations. Perhaps the highest priority is to improve understanding of the link between infection prevalence and clinical burden in both *P. vivax* mono-endemic settings and where it is coendemic with *P. falciparum*. Official estimates of national and regional disease burdens for *P. vivax* remain reliant on routine case reporting of unknown fidelity and are only crudely distinguished from *P. falciparum* [100]. It is illuminating that only 53 of the 95 *P. vivax* endemic countries were able to provide vivax-specific routine case reporting data, and there is a clear mandate for strengthening the routine diagnosis and reporting of *P. vivax* cases. Cartographic approaches to estimating *P. vivax* burden can therefore play a crucial role in triangulating with these estimates to provide insight into the distribution of the disease independent of health system surveillance and its attendant biases [27], [101]–[105]. There is also a particular need to define burden and clinical outcomes associated with *P. vivax* in pregnancy [9], [106] and other clinically vulnerable groups, most notably young children. Linking infection prevalence to clinical burden implies the need to better understand the contribution of relapsing infections to disease. Whilst the magnitude of this contribution is known to be highly heterogeneous, its geographical pattern is poorly measured and causal factors only partially understood [39], [41].

Further challenges lie in understanding how *P. falciparum* and *P. vivax* interact within human hosts and how these interactions manifest at population levels. Comparison of the maps for each species reveals a complete spectrum from areas endemic for only one parasite through to others where both species are present at broadly equal levels. Whilst identifying these patterns of coendemicity is an important first step, the implications in terms of risks of coinfection and clinical outcomes, antagonistic mechanisms leading to elevated severe disease risk, or cross-protective mechanisms of acquired immunity remain disputed [20], [107]–[109].

### Conclusions

To meet international targets for reduced malaria illness and death, and to progress the cause of regional elimination, the malaria research and control communities can no longer afford to neglect the impact of *P. vivax*. Its unique biology and global ubiquity present challenges to its elimination that greatly surpass those of its higher-profile cousin, *P. falciparum*. Making serious gains against the disease will require substantive strengthening of the evidence base on almost every aspect of its biology, epidemiology, control and treatment. The maps presented here are intended to contribute to this effort. They are all made freely available from the MAP website [110] along with regional and individual maps for every malaria-endemic country. Users can access individual map images or download the global surfaces for use in a geographical information system, allowing them to integrate this work within their own analyses or produce bespoke data overlays and displays. We will also make available, where permissions have been obtained, all underlying *P. vivax* parasite rate surveys used in this work.

## Supporting Information

Protocol S1 **Updating the global spatial limits of** ***Plasmodium vivax*** **malaria transmission for 2010.** S1.1 Overview. S1.2 Identifying Countries Considered *P. vivax* Malaria Endemic. S1.3 Updating National Risk Extents with *P. vivax* Annual Parasite Incidence Data. S1.4 Biological Masks of Transmission Exclusion. S1.5 Risk Modulation Based on Medical Intelligence. S1.6 Assembling the *P. vivax* Spatial Limits Map. S1.7 Refining Regions of Unstable Transmission after MBG Modelling. S1.8 Predicting Populations at Risk of *P. vivax* in 2010.
(DOC)

Protocol S2 **The Malaria Atlas Project** ***Plasmodium vivax*** **parasite prevalence database.** S2.1 Assembling the *Pv*PR Data. S2.2 Database Fidelity Checks. S2.3 Data Exclusions. S2.4 The *Pv*PR Input Data Set. S2.5 Age-Standardisation. S2.6 Regionalisation.
(DOC)

Protocol S3 **Bayesian model-based geostatistical framework for predicting** ***Pv*** **PR_1–99_.** S3.1 Bayesian Inference. S3.2 Model Overview. S3.3 Formal Presentation of Model.
(DOC)

Protocol S4 **Model validation procedures and additional results.** S4.1 Creation of Validation Sets. S4.2 Procedures for Testing Model Performance. S4.3 Validation Results.
(DOC)
