# Supplementary material for: A Long Neglected World Malaria Map: Plasmodium vivax Endemicity in 2010
Source: PLoS Negl Trop Dis. 2012 Sep 6;6(9):e1814. doi: 10.1371/journal.pntd.0001814 (PMC3435256; doi:10.1371/journal.pntd.0001814)
Supplement: Protocol S1 — Updating the global spatial limits of Plasmodium vivax malaria transmission for 2010. S1.1 Overview. S1.2 Identifying Countries Considered P. vivax Malaria Endemic. S1.3 Updating National Risk Extents with P. vivax Annual Parasite Incidence Data. S1.4 Biological Masks of Transmission Exclusion. S1.5 Risk Modulation Based on Medical Intelligence. S1.6 Assembling the P. vivax Spatial Limits Map. S1.7 Refining Regions of Unstable Transmission after MBG Modelling. S1.8 Predicting Populations at Risk of P. vivax in 2010. (DOC) [file pntd.0001814.s001.doc]

**Protocol S1 - Updating the global spatial limits of *Plasmodium vivax* malaria transmission for 2010**

**S1.1 Overview**

We have previously undertaken an exercise to define the geographical limits of *P. vivax* transmission in the year 2009 , stratifying the world into areas considered risk-free or at-risk of unstable (characterised by annual incidence less than 0.1‰) or stable (annual incidence exceeding 0.1‰) transmission. The components used to generate these classifications are (i) an initial identification of those countries housing autochthonous transmission within their borders (the *P. vivax* malaria endemic countries, *Pv*MECs); (ii) sub-nationally reported incidence records from health management information systems (*P. vivax* annual parasite incidence data, *Pv*API); (iii) additional medical intelligence providing refined risk designations for specific regions such as islands or cities; (iv) exclusion of risk in areas where the local annual temperature regime cannot support transmission in an average year; and (v) further exclusion or downgrading of risk in areas where extreme aridity is likely to limit transmission. For the present study we have repeated this procedure in full to generate an updated version for 2010. In this supplementary document we detail the data assembly procedures, how the various components are combined, and the resulting limits definition.

**S1.2 Identifying Countries Considered *P. vivax* Malaria Endemic**

The first version of the *P. vivax* spatial limits map was developed upon a template consisting of 95 *Pv*MECs . This list of countries was confirmed as up-to-date for the current 2010 iteration using the same approach and sources of international travel and health guidelines described previously .

**S1.3 Updating National Risk Extents with *P. vivax* Annual Parasite Incidence Data**

*Pv*API Data Processing

The *Pv*API data by country were obtained from various sources (Table S1.1). The format in which these data were made available varied considerably between countries. Ideally, all data would have been available by administrative unit and by year, with each record representing the estimated population for the administrative unit and the number of confirmed autochthonous malaria cases by each human malaria parasite species, which would allow an estimation of species-specific API. These requirements were sometimes not fulfilled completely and a number of problems were encountered. First, population data by administrative unit were sometimes unavailable, in which case these data were sourced separately (using the methods described in S1.8) or extrapolated from preceding years to estimate *Pv*API. Second, not all API data were species-specific. In these cases, a parasite species ratio was inferred from alternative sources and applied to provide an estimate of species-specific API. For example, such a ratio was often available as a single national figure, in which case it was applied uniformly throughout the country. Third, although a differentiation between microspcopy-confirmed and suspected cases and between autochthonous and imported cases was often provided, in some cases it had to be assumed that the data referred to confirmed, autochthonous cases.

*Pv*API Data Summaries

Table S1.1 summarizes *Pv*API data characteristics for all *Pv*MECs for which these were available. *Pv*API data were not available for countries in the Africa+ region, with the exception of Djibouti, Namibia, Saudi Arabia, South Africa, Swaziland and Yemen. For Botswana, risk was constrained to northern districts based upon information from the travel and health guidelines consulted . For other countries in the Africa+ region, stable risk of *P. vivax* transmission was assumed to be present throughout their territories. In total, *Pv*API data were not available for 42 identified *Pv*MECs, all in Africa+, with the exception of Uzbekistan.

The majority of the *Pv*API data were obtained through personal communication with individuals and institutions linked to national malaria control activities in each country. These are cited in Table S1.1 and acknowledged on the MAP website (http://www.map.ox.ac.uk/acknowledgements/). The specific aim was to collate data for the four most recent years of reporting, ideally including 2010. For six countries the last year of reporting available was 2010. For three countries, 2009 was the last year of reporting available, whilst 2008 and 2009 were the last years available for 24 and nine countries, respectively. For Colombia, risk data could not be obtained after 2005. In terms of the length of the period of reporting, one year of data was available for 14 countries, two years for nine countries, three years for nine countries and four or more years for 22 countries (Table S1.1).

A total of 18 countries reported at ADMIN1 level and 29 at ADMIN2 level. For southern China, Myanmar, Nepal and Peru, data were available at ADMIN3 level. In central and northern China data were available at ADMIN1 level. Data for Namibia and Venezuela were a mixture of ADMIN1 and ADMIN2 levels. In total, 17,893 administrative units in 53 countries were populated with *Pv*API data (Table S1.1).

Mapping *Pv*API Data

In order to map *Pv*API data consistently, they were reconciled to the 2009 version of the Global Administrative Unit Layers (GAUL) data set, implemented by the Food and Agriculture Organization of the United Nations (FAO) within the EC FAO Food Security for Action Programme . In some cases this reconciliation was not straightforward given differences in national sub-divisions. In such cases, alternative sources and maps were used to guide adequate matching of *Pv*API data. For some countries, digital boundary files of the administrative sub-divisions corresponding to *Pv*API data were supplied. These countries were: Afghanistan, Indonesia, Myanmar, Papua New Guinea, Peru, Solomon Islands, South Africa and Vietnam. In these cases, coastlines remained the same as the supplied shape files whilst borders between countries were made congruent with those in the GAUL dataset.

Classification of risk based on *Pv*API data was done as described previously . Classifications of extremely low, unstable transmission of *P. vivax* were assigned to administrative units reporting *Pv*API of less than 0.1 cases per 1,000 population per annum (p.a.), and those reporting a *Pv*API of ≥ 0.1 cases per 1,000 population p.a. were classified as having stable transmission.

**S1.4 Biological Masks of Transmission Exclusion**

We adopt our earlier approach to excluding areas at risk based on environmental non-suitability for *P. vivax* transmission . For completeness, we describe in full here the rationale and methods used.

Temperature Mask

In some regions, ambient temperature plays a key role in suppressing or precluding *P. vivax* transmission *via* various effects on stages of the parasite and *Anopheles* vector life cycles - most importantly by modulating the duration of the extrinsic incubation period of the parasite within the vector and by affecting daily survival rates of the latter . Here, we employ an existing model that evaluates temperature effects dynamically through time to generate for each pixel an index of temperature suitability proportional to vectorial capacity, an established biological metric of potential transmission intensity .

In brief, synoptic mean, maximum, and minimum monthly temperature records from 30-arcsec (~1×1 km) spatial resolution climate surfaces were converted to a continuous time series using spline interpolation. This represented the mean temperature profile across an average year. Diurnal variation was incorporated by adding a sinusoidal component to the time series with a wavelength of 24 hours and the amplitude driven by the difference between the spline-smoothed monthly minimum and maximum values. Ambient temperature can limit or preclude malaria transmission *via* a number of influences on components of the transmission cycle. Although temperature effects have been described on the survival and emergence rates of mosquito larvae , and vector feeding rates , the limiting effects of temperature on transmission are most pronounced in the interaction between vector lifespan and the duration of sporogony: the extrinsic incubation period during which the parasite matures into the sporozoite life stage within the vector. For *P. vivax* transmission to be biologically feasible, a cohort of anopheline vectors infected with the parasite must survive long enough for sporogony to complete within their lifetime. We modelled daily vector survival rate as a continuous function of local temperature regimes within each pixel using an established relationship drawn from a series of observational and modelling studies . Maximum vector lifespan was defined as 31 days since estimates of the longevity of the main dominant vectors indicate that 99% of anopheline vectors die in less than a month. The exceptions were areas that support the longer-lived *Anopheles sergentii* and *An. superpictus*, where 62 days were more appropriate . Sporogony is also strongly dependent on ambient temperature, so the time required for its completion varies continuously as temperatures fluctuate across a year . The dependence of sporogony duration on temperature is classically expressed using a simple temperature-sum model in which sporogony occurs after a fixed number of degree-days over a minimum temperature threshold for development.

The interaction between vector life span and sporogony duration was modelled for each pixel based on an assumption of constant vector emergence and the continuous evaluation of the expressions for daily vector survival and accumulation of degree days towards sporogony. A system of difference equations was implemented that, in effect, simulated the emergence of successive vector cohorts throughout the year, their declining population size as a function of temperature, and whether any constituent vectors survived long enough to complete sporogony. Those pixels in which no window existed across the year for the completion of sporogony were classified as being at zero risk of transmission. The temperature mask resulting from this process is shown in Figure S1.1.

Aridity Mask

A second driver of environmental suitability for *P. vivax* transmission is the availability of moisture. We adopted our earlier approach to mapping those areas where extreme aridity is likely to prevent transmission by restricting vector survival and availability of oviposition sites . Such areas were identified using pixels defined as 'bare areas' by the GlobCover land-cover classification product (ESA/ESA GlobCover Project, led by MEDIAS-France/POSTEL) . GlobCover products are derived from data provided by the Medium Resolution Imaging Spectrometer (MERIS), on board the European Space Agency’s (ESA) ENVIronmental SATellite (ENVISAT), for the period between December 2004 and June 2006, and are available at a spatial resolution of 300 meters . This layer was first resampled to a 1×1 km grid using a majority filter, and all pixels classified as “bare areas” by GlobCover were overlaid onto the *Pv*API surface. The result is shown in Figure S1.2. The aridity mask was treated differently from the temperature mask to allow for the possibility of the adaptation of human and vector populations to arid environments . A more conservative approach was taken whereby risk was down-regulated by one class. In other words, GlobCover’s bare areas defined originally as at stable risk by *Pv*API were stepped down to unstable risk and those classified initially as unstable were classed as malaria free.

**S1.5 Risk Modulation Based on Medical Intelligence**

We adopt the same data sources and assembly protocols for incorporation of medical intelligence information as described previously . For completeness, we detail these data and methods again here.

Urban Transmission

Urban areas are less malarious than the surrounding rural environments due to the distinct ecological conditions presented by man-made environments . The extent to which transmission is reduced will vary according to the local *Anopheles* species. Urbanisation has been shown to reduce malaria transmission, measured by the entomological inoculation rate, by an order of magnitude across Africa, due to reduced vector diversity and density, as well as lower anopheline survival, biting and sporozoite rates in urban versus rural areas . *Anopheles darlingi*, the main malaria vector in America, has shown itself to be similarly unsuited to urban environments .

Urban malaria transmission is more entrenched in the Indian subcontinent because of the presence of *An. stephensi* and, to a lesser extent, *An. culicifacies*, both recognised urban malaria vectors . No malaria vector is better adapted to urban environments than *An. stephensi*, and this is due to its ability to breed in all types of artificial collections of water, such as wells, pits, tanks and drains . *Anopheles culicifacies* is less resilient to man-made environments and is particularly affected by pollution of water sources . Importantly, the vector densities and sporozoite rates of both these species have been shown to decrease from peri-urban to urban areas . Despite this decrease, it is estimated that approximately 8% of reported malaria cases in India come from urban areas , with incidence often surpassing the stable risk threshold. Reported annual parasite incidence (API) estimates amongst 86 cities across India in 1993 ranged from 0 to 51.85 cases per 1,000 people per annum (p.a.), with a median of 0.97 . Seventy of these cities would have been classified as supporting stable transmission according to the API threshold used in this paper (i.e. API ≥0.1 case per 1,000 people p.a.). In 2004, the API in an impoverished area located in the outskirts of Kolkata was measured at 1.5 cases per 1,000 residents p.a., with the majority (97%) due to *P. vivax* . Since *An. culicifacies* seems to be more affected by the process of urbanisation, it was assumed that urban malaria transmission is maintained mainly by *An. stephensi* (Figure S1.4) as defined by the rules of risk modulation described below.

Risk Modulation in Specified Urban Areas

There are 59 cities cited as being malaria free in the two sets of international travel and health guidelines consulted (Table 1). In addition, urban areas in China, the Philippines and Indonesia (specifically those located in Sumatra, Kalimantan, Nusa Tenggara Barat and Sulawesi) are said to be malaria free. This is obviously not a comprehensive list of malaria free cities but rather one restricted to main destinations of interest to travellers. Specific cities were geo-positioned and their urban extents were identified using the Global Rural Urban Mapping Project (GRUMP) urban extents layer . In China, the Philippines and specified areas of Indonesia all urban extents were identified and mapped. The resulting layer was overlaid on the *Pv*API layer and biological masks to identify the underlying risk of malaria. Those cities falling within the range of *An. stephensi* were also identified.

Of the 59 specified cities, 17 are in areas where *P. vivax* malaria transmission is absent as defined by the *Pv*API layer and the biological masks (e.g. highland areas). The urban extents of the remaining 42 cities cover areas defined as unstable or stable transmission or both (Table S1.2). Only eight of these cities fall within the range of *An. stephensi*: six in India (Bangalore, Kolkata, Mumbai, Nagpur, Nashik and Pune) and two in Myanmar (Mandalay and Yangon; Figure). In addition, cities in south-western Yunnan, China, also fall in areas inhabited by this vector.

For all cities falling outside the range of *An. stephensi*, risk was classified as absent throughout their urban extents. For those cities falling within the range of *An. stephensi*, risk was assumed to be one level lower than the surrounding risk defined by *Pv*API data and the biological masks. This is to allow for the potential transmission of malaria by *An. stephensi* combined with the transmission reducing effects of urban areas .

Risk Exclusion in Administrative Areas

Some sub-national administrative areas and territories are listed as being malaria free by the international travel and health guidelines consulted . These are shown in Table 2. Such territories were mapped using the GAUL data set and risk within them was assigned a malaria free category, if not already classified as such by the *Pv*API layer and the biological masks. In addition to the territories listed in Table S1.3, the island of Socotra, in Yemen, has not reported cases since 2005 after malaria elimination activities were initiated in 2000 ; this island was assumed to be malaria free. Two further exclusions were those of the island of Aneityum, in Vanuatu , and the Angkor Watt area, in Cambodia (corresponding to two districts in Siem Reap province), which were classified as malaria free following personal communication with malaria experts in these countries.

**S1.6 Assembling the *P. vivax* Spatial Limits Map**

Figure S1.3 summarises the different steps undertaken to assemble the *P. vivax* spatial limits map. The various data sources described above were progressively applied on a geographical information system with subsequent reductions in estimated area and population at risk. This sequence is illustrated as different maps in Figure S1.5A-E

**S1.7 Refining Regions of Unstable Transmission after MBG Modelling**

In some regions within the estimated limits of stable transmission, the model-based geostatistical predictions of *Pv*PR­_1-99_­ were extremely low, either because of a dense abundance of survey data reporting zero infections or, in Africa, because of very high coincident Duffy negativity phenotype frequencies suppressing prevalence predictions. Such areas are not appropriately described as at stable risk and so we defined a decision rule whereby pixels predicted with high certainty (probability >0.9) of being less than 1% *Pv*PR_1-99_ were identified and assigned to the unstable class, thereby modifying the original transmission limits (Figure S1.5F). These augmented mapped limits were combined with a 2010 population surface derived from the Global Rural Urban Mapping Project (GRUMP) *beta* version to estimate the number of people living at no risk or at unstable or stable risk within each country and region.

**S1.8 Predicting Populations at Risk of *P. vivax* in 2010**

The Global Rural Urban Mapping Project (GRUMP) *beta* version provides gridded population counts and population density estimates at 1×1 km spatial resolution for the years 1990, 1995 and 2000, both adjusted and unadjusted to the United Nations’ national population estimates . The adjusted population counts for the year 2000 were projected to 2010 by applying the relevant urban and rural national growth rates by country using methods described previously . The urban growth rates were applied to populations residing within the GRUMP-defined urban extents , and the rural rates were applied elsewhere. National 2010 totals were then adjusted to match those estimated by the United Nations .

The population grid was overlaid with the categorised limits map and the total population located within each limits class was computed. An equivalent calculation was made of the land area associated with each class, by replacing the population grid with one quantifying the surface area of each pixel, taking into account the equirectangular map projection used. Additionally, the population grid was combined with the uncertainty maps to provide a population-weighted index of uncertainty (the product of the log of population density and the reciprocal of the probability of correct class assignment).

**Table S1.1**. Summary of the *P. vivax* annual parasite incidence (*Pv*API) data assembled for each country. The data are grouped by the three global regions defined by Hay *et al.* : Africa+, America and Central and South East (CSE) Asia. ADMIN1, 2 or 3 refers to the administrative division level (first, second or third level) at which data were available. The number of risk units refers to how many administrative units, at the level specified, were populated with actual data. Year start and Year end mark the start and end of the period for which data were available.

| **Region** | **Country** | **Admin. level** | **Risk units** | **Year start** | **Year end** | **Source** |
| --- | --- | --- | --- | --- | --- | --- |
| Africa+ | Djibouti | ADMIN1 | 5 | 2007 | 2009 |  |
| Africa+ | Namibia | ADMIN1 & ADMIN2 | 30 | 2009 | 2009 |  |
| Africa+ | Saudi Arabia | ADMIN1 | 13 | 2005 | 2006 |  |
| Africa+ | South Africa | ADMIN2 | 257 | 2006 | 2009 |  |
| Africa+ | Swaziland | ADMIN2 | 53 | 2007 | 2009 |  |
| Africa+ | Yemen | ADMIN1 | 19 | 2002 | 2006 |  |
| America | Argentina | ADMIN2 | 513 | 2008 | 2008 |  |
| America | Belize | ADMIN1 | 6 | 2006 | 2006 |  |
| America | Bolivia | ADMIN2 | 113 | 2008 | 2008 |  |
| America | Brazil | ADMIN2 | 5510 | 2004 | 2008 |  |
| America | Colombia | ADMIN2 | 1087 | 2005 | 2005 |  |
| America | Costa Rica | ADMIN2 | 81 | 2006 | 2006 |  |
| America | Ecuador | ADMIN2 | 220 | 2005 | 2008 |  |
| America | El Salvador | ADMIN1 | 14 | 2006 | 2006 |  |
| America | French Guiana | ADMIN2 | 21 | 2006 | 2006 |  |
| America | Guatemala | ADMIN1 | 22 | 2006 | 2006 |  |
| America | Guyana | ADMIN1 | 10 | 2004 | 2007 |  |
| America | Honduras | ADMIN2 | 291 | 2005 | 2008 |  |
| America | Mexico | ADMIN2 | 2454 | 2005 | 2008 |  |
| America | Nicaragua | ADMIN1 | 17 | 2004 | 2007 |  |
| America | Panama | ADMIN2 | 68 | 2006 | 2007 |  |
| America | Paraguay | ADMIN2 | 219 | 2008 | 2008 |  |
| America | Peru | ADMIN3 | 1828 | 2005 | 2008 |  |
| America | Suriname | ADMIN1 | 10 | 2008 | 2008 |  |
| America | Venezuela | ADMIN1 & ADMIN2 | 30 | 2004 | 2008 |  |
| CSE Asia | Afghanistan | ADMIN2 | 398 | 2005 | 2008 |  |
| CSE Asia | Azerbaijan | ADMIN1 | 73 | 2005 | 2008 |  |
| CSE Asia | Bangladesh | ADMIN2 | 64 | 2007 | 2008 |  |
| CSE Asia | Bhutan | ADMIN1 | 20 | 2004 | 2010 |  |
| CSE Asia | Cambodia | ADMIN1 | 26 | 2005 | 2008 |  |
| CSE Asia | China | ADMIN1 & ADMIN3 | 263 | 2003 | 2007 |  |
| CSE Asia | Georgia | ADMIN2 | 79 | 2005 | 2010 |  |
| CSE Asia | India | ADMIN2 | 574 | 2004 | 2007 |  |
| CSE Asia | Indonesia | ADMIN2 | 346 | 2005 | 2008 |  |
| CSE Asia | Iran | ADMIN2 | 283 | 2007 | 2008 |  |
| CSE Asia | Iraq | ADMIN2 | 11 | 2005 | 2008 |  |
| CSE Asia | Lao PDR | ADMIN2 | 139 | 2006 | 2008 |  |
| CSE Asia | Kyrgyzstan | ADMIN1 | 1 | 2008 | 2008 |  |
| CSE Asia | Malaysia | ADMIN1 | 15 | 2003 | 2010 |  |
| CSE Asia | Myanmar | ADMIN3 | 325 | 2006 | 2008 |  |
| CSE Asia | Nepal | ADMIN3 | 75 | 2005 | 2010 |  |
| CSE Asia | Pakistan | ADMIN2 | 119 | 2005 | 2008 |  |
| CSE Asia | Papua New Guinea | ADMIN2 | 87 | 2005 | 2007 |  |
| CSE Asia | Philippines | ADMIN2 | 82 | 2004 | 2007 |  |
| CSE Asia | Rep. of Korea | ADMIN2 | 239 | 2005 | 2008 |  |
| CSE Asia | Solomon Islands | ADMIN1 | 10 | 2003 | 2007 |  |
| CSE Asia | Sri Lanka | ADMIN2 | 25 | 2006 | 2010 |  |
| CSE Asia | Tajikistan | ADMIN2 | 56 | 2005 | 2008 |  |
| CSE Asia | Thailand | ADMIN1 | 76 | 2006 | 2010 |  |
| CSE Asia | Timor-Leste | ADMIN1 | 13 | 2008 | 2008 |  |
| CSE Asia | Turkey | ADMIN2 | 926 | 2008 | 2008 |  |
| CSE Asia | Vanuatu | ADMIN1 | 6 | 2003 | 2007 |  |
| CSE Asia | Viet Nam | ADMIN2 | 671 | 2005 | 2008 |  |

**Table S1.2** Cities cited as being malaria free by the sources consulted . Defined risk refers to the malaria risk categories defined by the *Pv*API layer and biological masks; note that urban extents often cover more than one category. Modified risk refers to the new malaria risk categories assigned according to the rules described in the text. Cities where the defined risk was “free” were not affected by these rules.

| **Country** | **City** | **Defined risk** | **Modified risk** |
| --- | --- | --- | --- |
| Azerbaijan | Baku | Free, unstable | Free |
| Bangladesh | Dhaka | Free | - |
| Belize | Belize | Unstable | Free |
| Bolivia | La Paz | Free | - |
| Botswana | Gaborone | Free | - |
| Cambodia | Phnom Penh | Free, unstable | Free |
| Colombia | Bogota | Free, unstable | Free |
| Colombia | Cartagena | Free, unstable | Free |
| Costa Rica | Puerto Limon | Unstable | Free |
| Ecuador | Guayaquil | Unstable, stable | Free |
| Ecuador | Quito | Free | - |
| Eritrea | Asmara | Stable | Free |
| Ethiopia | Addis Ababa | Stable, free | Free |
| French Guiana | Cayenne | Free | - |
| Georgia | Tblisi | Unstable, free | Free |
| Guatemala | Antigua | Free | - |
| Guatemala | Guatemala | Free | - |
| Honduras | San Pedro Sula | Unstable | Free |
| Honduras | Tegucigalpa | Unstable, free | Free |
| India | Bangalore | Stable | Unstable |
| India | Kolkata | Unstable, stable | Free, unstable |
| India | Mumbai | Stable, unstable | Unstable, free |
| India | Nagpur | Stable | Unstable |
| India | Nasik | Unstable | Free |
| India | Pune | Unstable | Free |
| Indonesia | Jakarta | Free | - |
| Iraq | Baghdad | Free | - |
| Iraq | Ramadi | Free | - |
| Iraq | Tikrit | Free | - |
| Kenya | Nairobi | Stable | Free |
| Kyrgyzstan | Bishkek | Free | - |
| Laos | Vientiane | Free, unstable | Free |
| Myanmar | Mandalay | Free, stable, unstable | Free, unstable |
| Myanmar | Yangon | Unstable | Free |
| Nepal | Kathmandu | Free | - |
| Nicaragua | Managua | Unstable | Free |
| Panama | Panama | Unstable | Free |
| Peru | Cuzco | Free | - |
| Saudi Arabia | Jeddah | Unstable | Free |
| Saudi Arabia | Mecca | Unstable | Free |
| Saudi Arabia | Medina | Unstable | Free |
| Saudi Arabia | Riyadh | Free | - |
| Saudi Arabia | Ta'if | Unstable | Free |
| Suriname | Paramaribo | Free | - |
| Thailand | Bangkok | Free, unstable | Free |
| Thailand | Chiang Mai | Stable | Free |
| Thailand | Chiang Rai | Unstable | Free |
| Thailand | Koh Phangan | Stable | Free |
| Thailand | Koh Samui | Stable | Free |
| Thailand | Pattaya | Unstable | Free |
| Viet Nam | Can Tho | Free, unstable | Free |
| Viet Nam | Da Nang | Unstable | Free |
| Viet Nam | Haiphong | Free | - |
| Viet Nam | Hanoi | Free, unstable | Free |
| Viet Nam | Ho Chi Minh City | Unstable | Free |
| Viet Nam | Hue | Free, unstable | Free |
| Viet Nam | Nha Trang | Free, unstable | Free |
| Viet Nam | Qui Nhon | Unstable | Free |
| Yemen | Sana’a | Unstable | Free |

**Table S1.3.** Administrative areas defined as being malaria free by international travel and health guidelines.

| **Country** | **Administrative areas/sub-national territories** |
| --- | --- |
| Ecuador | Galapagos |
| French Guiana | Devil's Island |
| Mauritania | Adrar, Dakhlet-Nouadhibou, Inchiri and Tiris-Zemmour regions |
| Philippines | Aklan, Albay, Benguet, Bilaran, Bohol, Camiguin, Capiz, Catanduanes, Cavite, Cebu, Guimaras, Iloilo, Northern Leyte, Southern Leyte, Marinduque, Masbate, Eastern Samar, Northern Samar, Western Samar, Sequijor, Sorsogon, Surigao Del Norte and metropolitan Manila |
| Sri Lanka | Colombo, Galle, Gampaha, Kalutara, Matara, and Nuwara Eliya |
| Venezuela | Margarita Island (Nueva Esparta) |

**
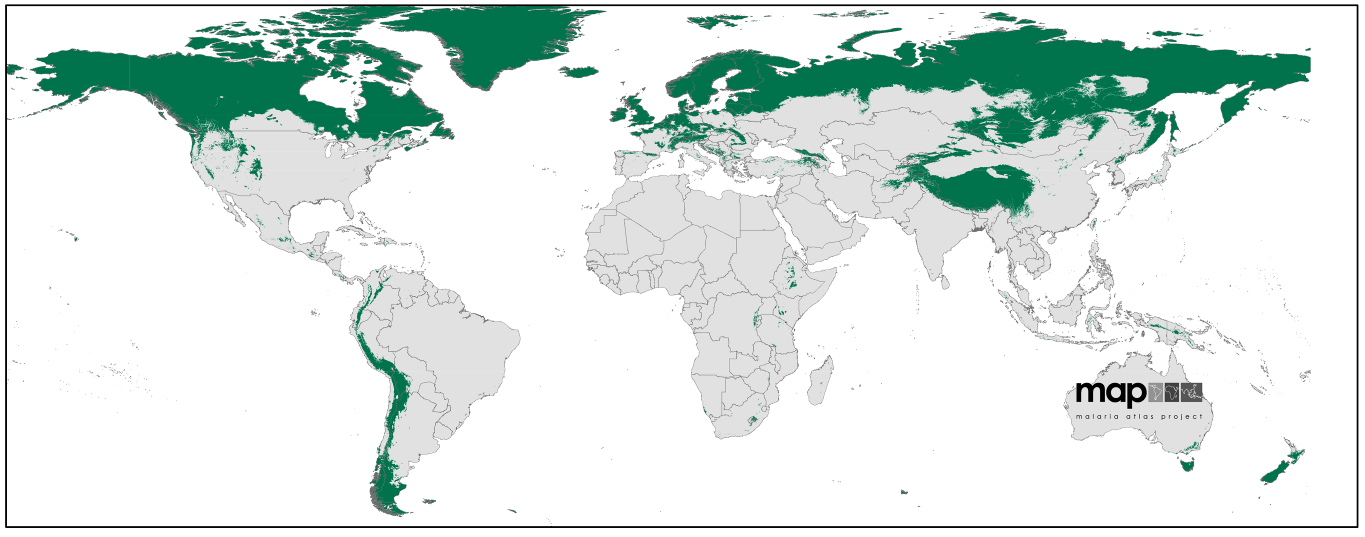
**

**Figure S1.1. Environmental suitability for transmission of *P. vivax* as defined by temperature.** Areas shaded green are those in which no windows exist across an average year in which the annual temperature regime is likely to support the presence of infectious vectors. The temperature suitability model is described in full elsewhere .

**
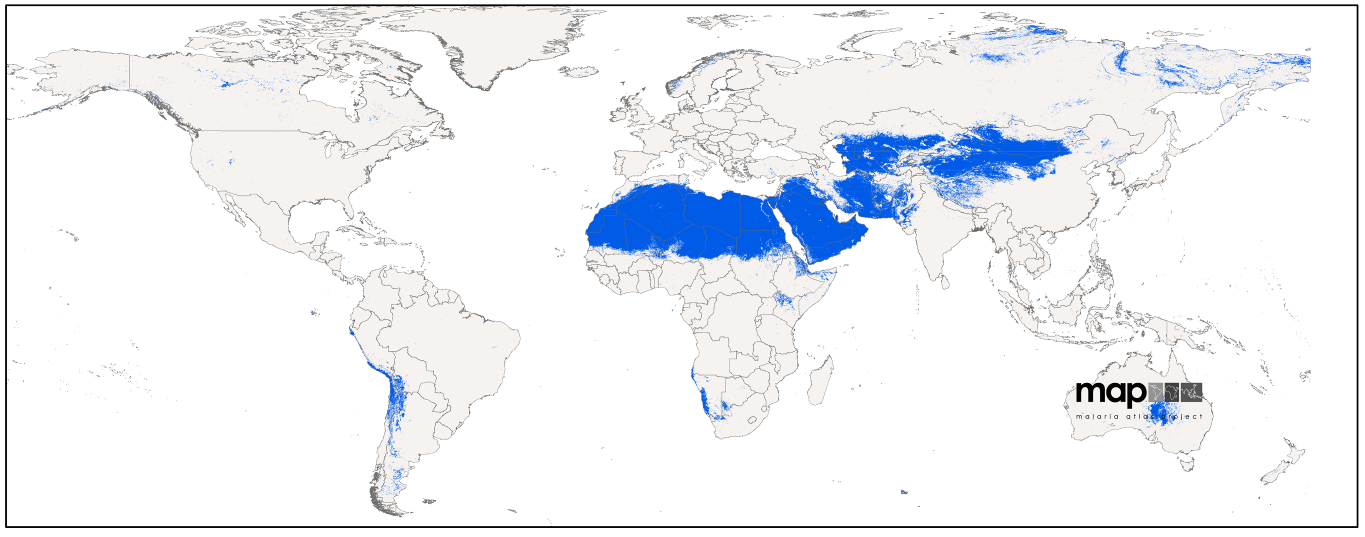
**

**Figure S1.2. Environmental suitability for transmission of *P. vivax* as defined by extreme aridity.** Areas shaded grey are those classified as bare areas by the GlobCover land cover product, interpreted as lacking sufficient moisture to support populations of *Anopheles* necessary for transmission.

**
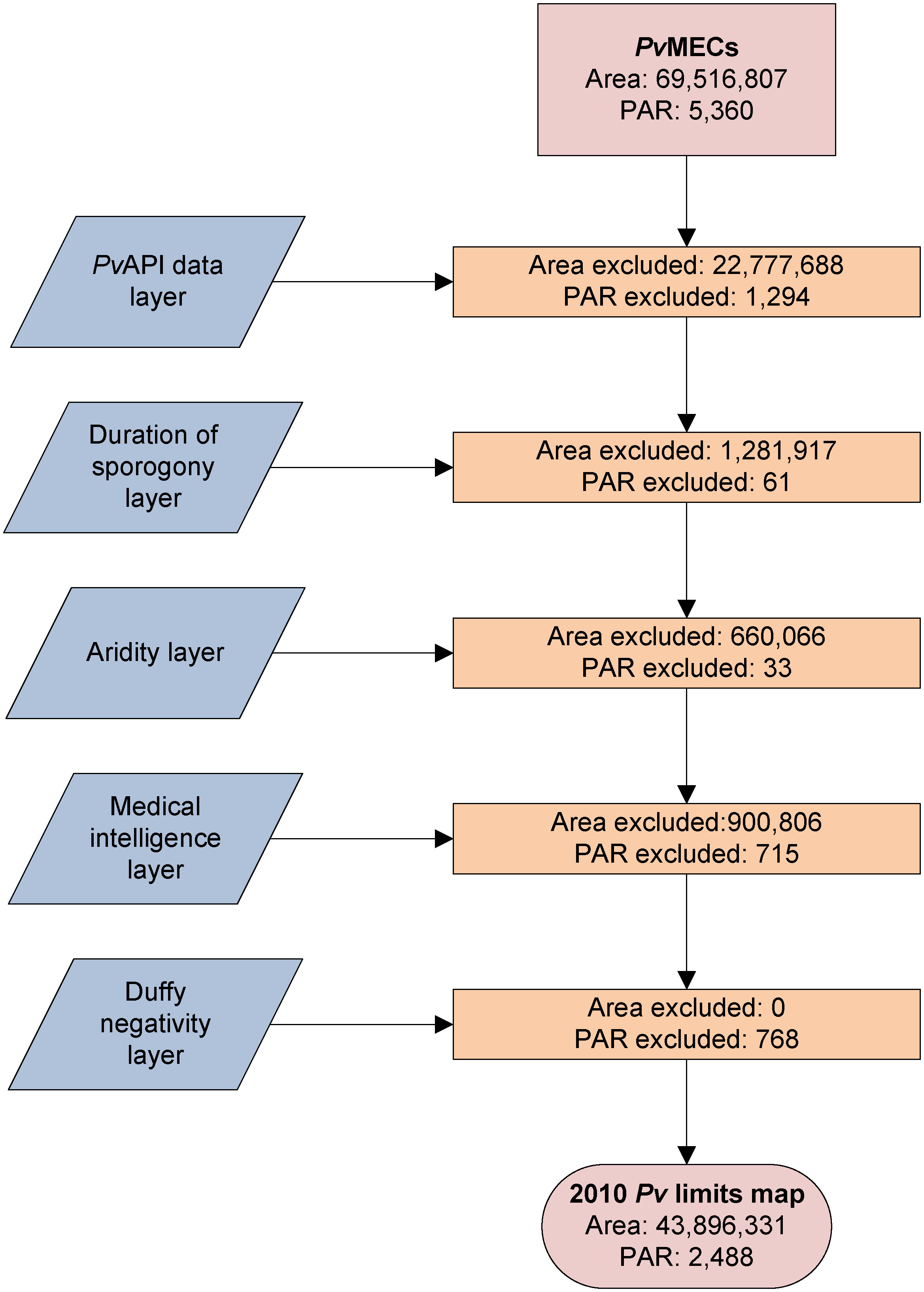
**

**Figure S1.3.** **Flow chart of the various exclusion layers used to derive the final map.** Area (expressed in km^2^) and population at risk (PAR; expressed in millions) excluded are shown at each step to illustrate how these were reduced progressively.


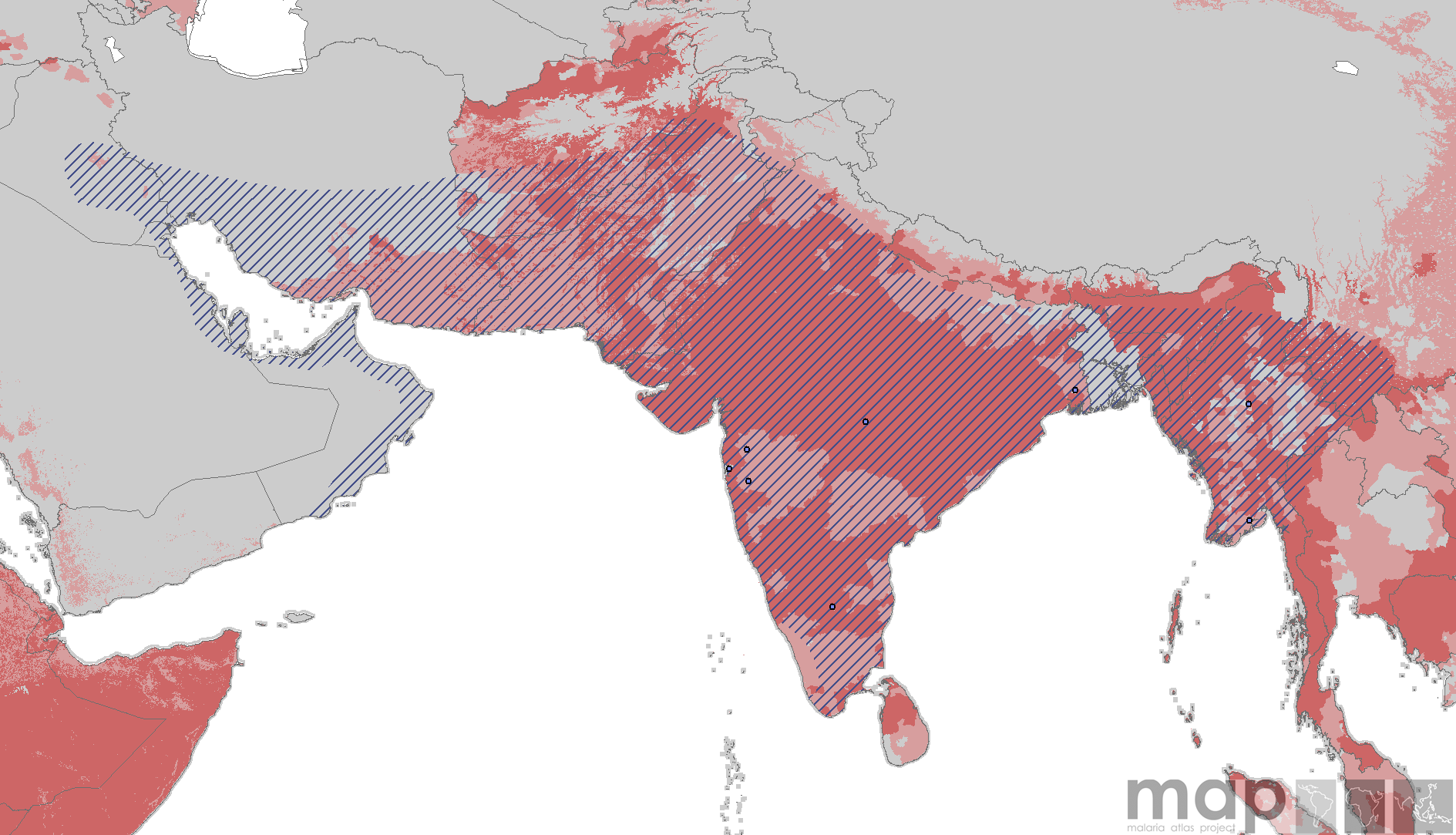


**Figure S1.4.** Distribution of *An. stephensi* (hatched area) and location of the eight cities falling within this range (light blue circles) overlaid on the *Pv*API/biological masks-defined *P. vivax* limits of transmission.

**
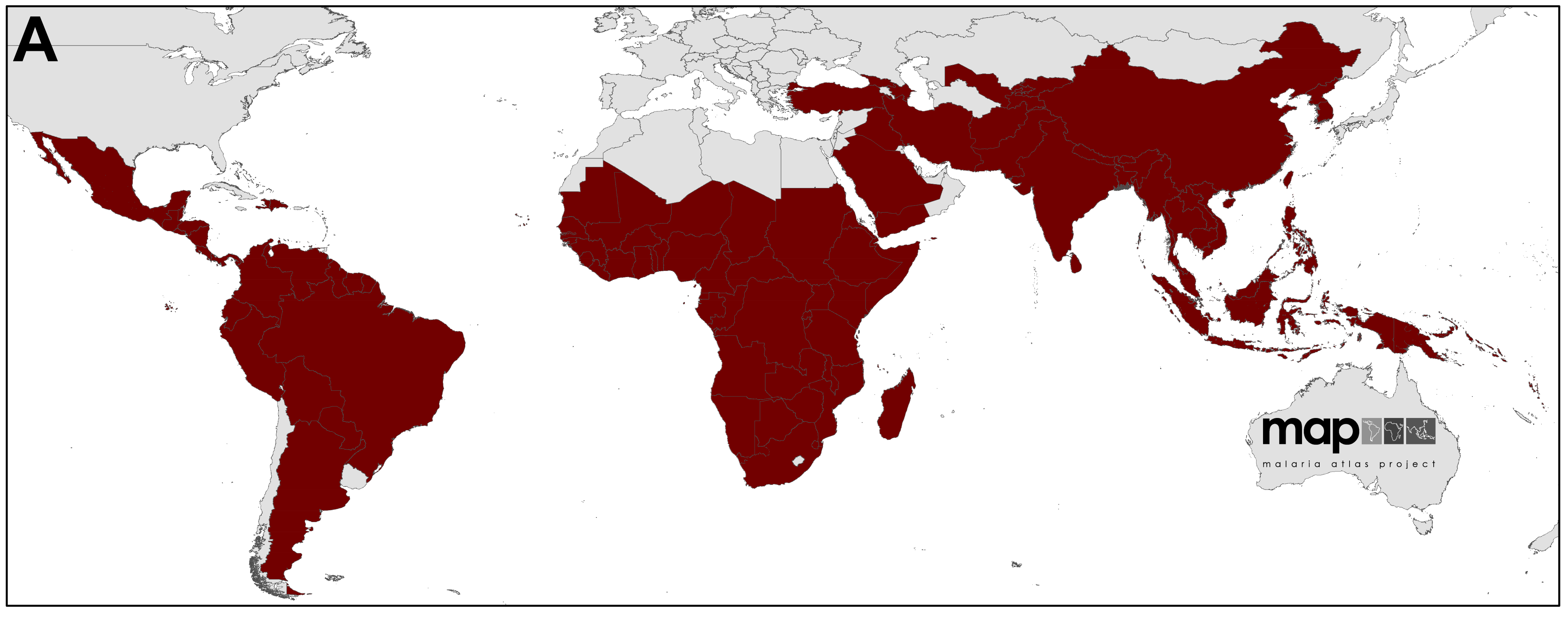
**

**
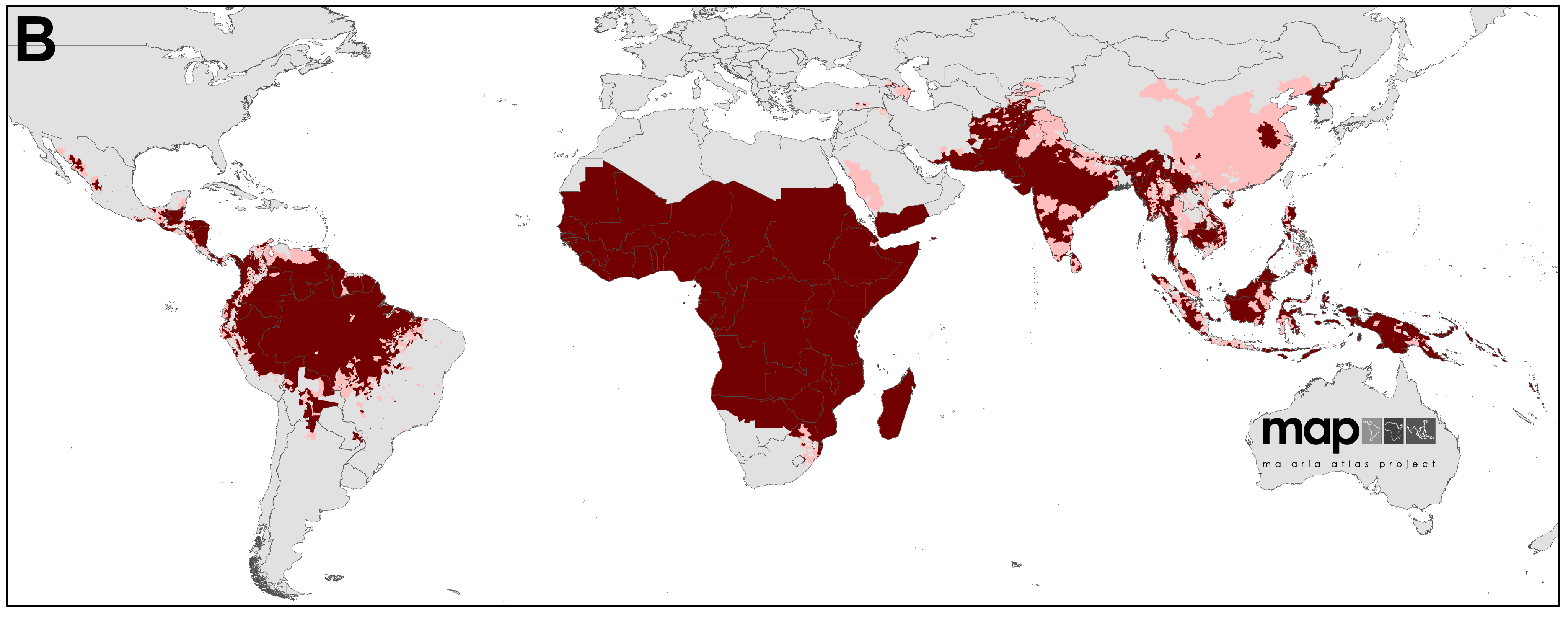
**

**
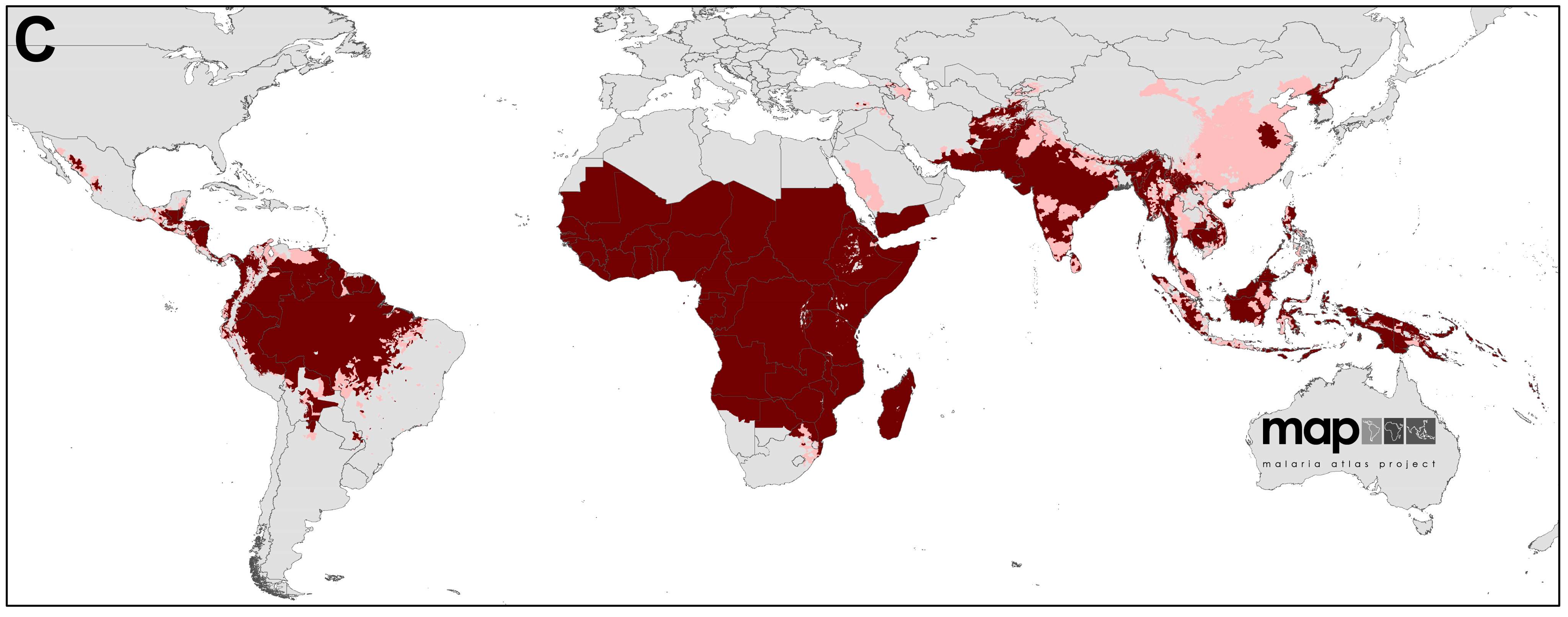
**

**
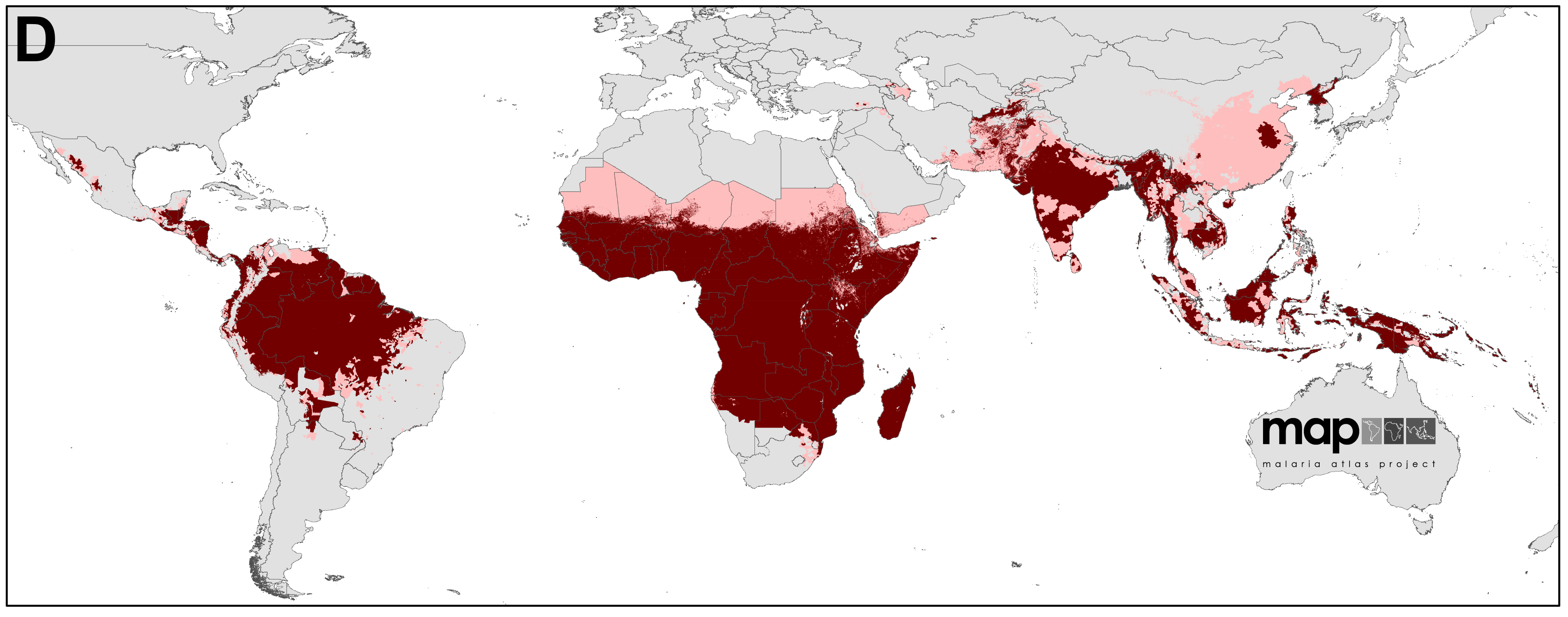
**

**
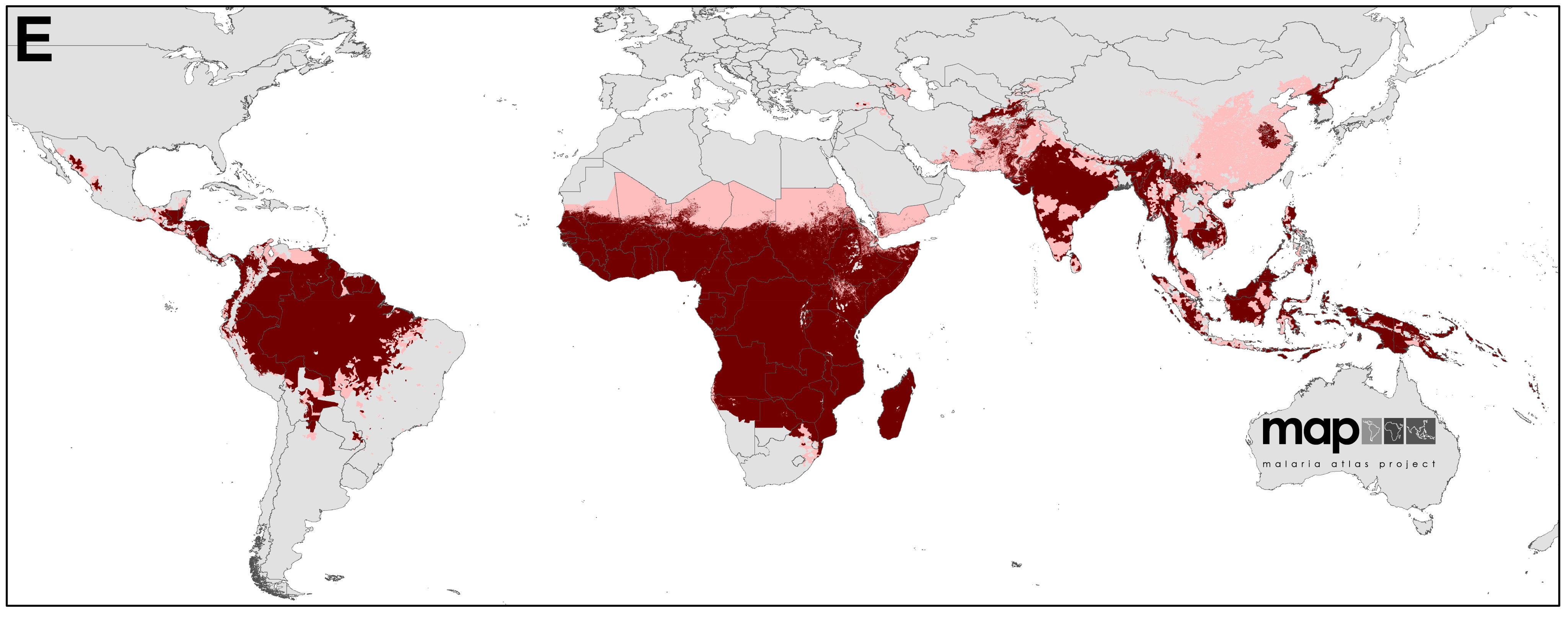
**

**
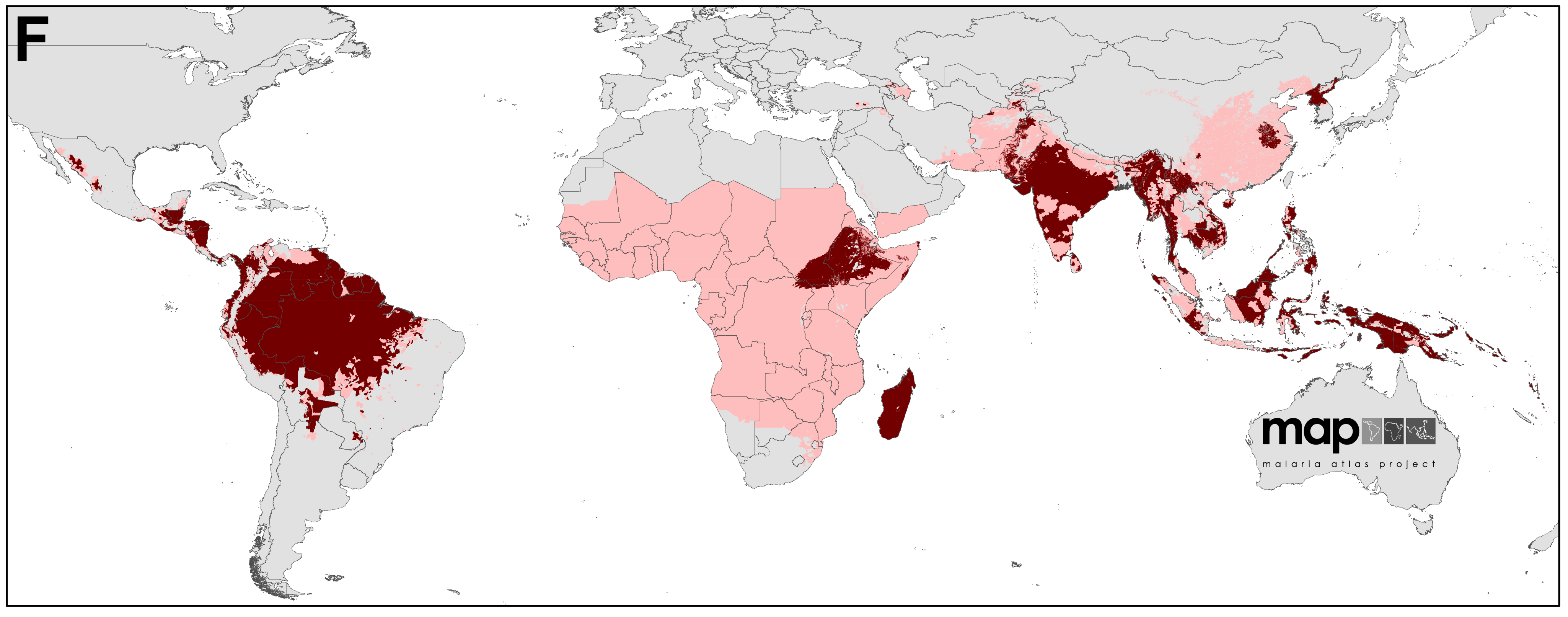
**

**Figure S1.5.** **Map sequence illustrating the different exclusion layers applied.** A = all regions of the 95 *P. vivax* endemic countries; B = downgrading or exclusion of risk informed by annual parasite incidence data (*Pv*API); C = additional exclusion of risk informed by the biological temperature mask; D = additional downgrading or exclusion of risk informed by the aridity mask; E = additional downgrading or exclusion of risk informed by medical intelligence and international travel and health guidelines; F = the final limits definition after additionally downgrading risk in stable areas predicted to have very low prevalence by the MBG model. Stable transmission is shown in red, unstable transmission in pink and *P. vivax* malaria free areas in grey.

**References**
