# Supplementary material for: A Long Neglected World Malaria Map: Plasmodium vivax Endemicity in 2010
Source: PLoS Negl Trop Dis. 2012 Sep 6;6(9):e1814. doi: 10.1371/journal.pntd.0001814 (PMC3435256; doi:10.1371/journal.pntd.0001814)
Supplement: Protocol S2 — The Malaria Atlas Project Plasmodium vivax parasite prevalence database. S2.1 Assembling the PvPR Data. S2.2 Database Fidelity Checks. S2.3 Data Exclusions. S2.4 The PvPR Input Data Set. S2.5 Age-Standardisation. S2.6 Regionalisation. (DOC) [file pntd.0001814.s002.doc]

**Protocol S2: The Malaria Atlas Project *Plasmodium vivax* parasite prevalence database**

The Malaria Atlas Project (MAP) database includes estimates of *Plasmodium falciparum* and *P. vivax* parasite prevalence derived from random community surveys covering the period 1985 to 2010 . This document describes the *P. vivax* parasite rate (*Pv*PR) data assembly, auditing steps performed on this database, the exclusions applied prior to modelling and some key features of the *Pv*PR data set used in the global *P. vivax* endemicity models described in this paper.

**S2.1 Assembling the *Pv*PR Data**

The *Pv*PR data inclusion criteria, search strategy, extraction and geo-positioning have been described in detail elsewhere . They are reviewed briefly in the following paragraphs.

*Pv*PR Data Inclusion Criteria

The inclusion criteria of the MAP *Pv*PR database are listed in Table S2.1. Two of these have been modified since the original protocols set out at the onset of MAP . First, the original inclusion criterion of a minimum sample size of ≥50 individuals surveyed was removed because the endemicity models adjust for sample size. A total of 2,243 small sample surveys (n<50) would have been otherwise excluded without this revision. Second, the 36 months duration interval between surveys conducted at the same location (spatial duplicates) was relaxed to three months.

Data Search Strategies

Data searches aimed at the retrieval of data from published and unpublished sources for as many *P. vivax* malaria endemic countries (*Pv*MECs) as possible. The published scientific literature was scanned weekly for data through subscription to malaria publications newsletters (mainly Malaria World newsletters (<http://www.malaria-world.com/>) and the Environmental Health at USAID malaria bulletins (<http://www.ehproject.org/>)). This was complemented by periodic data searches in online reference archives (mainly PubMed (<http://www.ncbi.nlm.nih.gov/sites/entrez>), ISI Web of Knowledge ([http://wok.mimas.ac.uk](http://wok.mimas.ac.uk/)) and Scopus ([http://www.scopus.com](http://www.scopus.com/))) to ensure that all relevant publications were captured. Keywords used in these searches were “malaria” and “[Country Name]”. Data from unpublished sources were mainly obtained through personal communication with malaria specialists. Full acknowledgement of these interactions and data provision is provided on the MAP website (<http://www.map.ox.ac.uk/acknowledgements>).

Data Abstraction and Entry

The minimum required data fields for each record were: description of the study area (name, administrative divisions and geographical coordinates, if available), the dates of start and end of the survey (month and year) and information about blood examination (number of individuals tested, number positive for *P. vivax* and examination method). Authors were contacted to request any missing information. Some survey results were reported as an aggregate in space (e.g. a single *Pv*PR for a group of villages) or time (e.g. a mean *Pv*PR estimated from four different surveys conducted in a period of time). In such cases, authors were contacted to attempt to obtain disaggregated data (e.g. a *Pv*PR estimate per village or per each of the cross-sectional surveys conducted in a time period). Data were entered in a PostgreSQL 8.3 database (PostgreSQL Global Development Group, 2009) running on a Unix platform.

Data Geo-Positioning

Data geo-positioning was a particularly demanding task during data entry. The same guidelines described previously were used here. According to their spatial representation, data were classified as points (corresponding to an area ≤10 km^2^), wide-areas (>10 and ≤25 km^2^), small polygons (>25 and ≤100 km^2^) or large polygons (>100 km^2^). Authors were contacted when reporting results as polygon data to try to disaggregate them into points or wide-areas. Various digital resources were used to geo-position data, amongst which the most useful were Microsoft Encarta Encyclopedia (Microsoft, 2004) and Google Earth (Google, 2009). Importantly, the provision of GPS readings accompanying raw data obtained through personal communication both decreased the burden of this task and improved the precision of data geo-positioning.

**S2.2 Database Fidelity Checks**

The entire database was first checked with a series of simple range-check constraint queries to identify potential errors that could have occurred during data entry. These queries assessed all data fields relevant to modelling for missing or inconsistent information. The fields checked included those describing the study area (area type, geographical coordinates, and urban or rural author definitions) and those providing specific information about the survey (month and year of start and end of the survey, age range of study population, number examined and positive for *P. vivax*, and diagnostic method utilised).

The second objective was to check that survey sites were located precisely with respect to the master raster grid templates in which the endemicity models were developed. The locations therefore needed to be on grid squares identified as land and within the border of the country in which the survey was conducted. All survey locations were intersected with the relevant grids and erroneous locations were identified and corrected manually. Typically this occurred in areas of complex coastlines with an average displacement of <1 km.

The final objective was to check for any duplicates introduced during the iterative data assembly process. Pairs of survey sites found within 1 km were listed and further checks identified whether they corresponded to the same location.

**S2.3 Data Exclusions**

The database was subjected to exclusions in order to attempt optimal spatial and temporal resolution of the data prior to modelling. Large (n=43) and small polygon data (n=41) were excluded because these records represented areas larger than the 5 × 5 km spatial resolution grid output of the model. In total, 226 records were excluded for the reasons outlined. Table S2.2 and Figure S2.1 summarise these exclusions by region. Spatiotemporal duplicates (i.e. those conducted at the same location with less than three months gap between surveys) were excluded unless it could be shown that different communities were sampled by the different surveys (e.g. separate but neighbouring schools).

**S2.4 The *Pv*PR Input Data Set**

The data exclusions outlined above resulted in the *Pv*PR input data set for the geo-statistical models. Some summary figures describing this data set are presented in Table S2.3 and are further discussed below.

On 25th November 2011, after all checks were performed, the database was considered ready for the endemicity models. *Pv*PR data were available for 52 countries (12 in America, 18 in Africa+ and 22 in CSE Asia) (Table S2.2). A total of 43 *Pv*MECs, mostly in Africa+, were not represented in the database. This explains the significantly lower number of *Pv*PR data records relative to the MAP *Pf*PR database . The *Pv*MECs outside Africa+ not represented in the database were Argentina, Azerbaijan, Belize, Bhutan, El Salvador, Georgia, Guyana, Iran, Korea DPR, Kyrgyzstan, Panama, Paraguay, Republic of Korea and Uzbekistan. The five data richest countries were Indonesia (n=4,457), Ethiopia (n=826), Viet Nam (n=657), Afghanistan (n=493) and Bangladesh (n=365). The *Pv*PR data were extracted from 432 different sources.

Total Number of Records

A total of 9,970 *Pv*PR estimates between 1985 and 2010 were available for modelling (388 in America, 1,640 in Africa+ and 7,942 in CSE Asia; Figures S2.1 and S2.2). There was a lack of *Pv*PR data from central, western and eastern Africa and 86% of the data from Africa+ derived from large surveys in three countries: Ethiopia, Sudan and Zambia. In CSE Asia, 56% of the data were from surveys done in one country, Indonesia. In the Americas, the majority of the data (45%) were from Brazil.

Summary of Recorded *Pv*PR Estimates

The recorded *Pv*PR values were generally low. Globally, more than half (51%) of the *Pv*PR surveys reported zero prevalence. In Africa+, 79% of the data were absence records, compared to 44% in America and 46% in CSE Asia. In Africa+, median and mean *Pv*PR were zero and 0.01, respectively. The *Pv*PR in America had a median of 0.01 and a mean of 0.03 and in CSE Asia the median was 0.01 and the mean 0.04 (Table S2.3).

Data and Geographic Coordinate Sources

The great majority of the *Pv*PR data (81%) were obtained from sources that had not been peer-reviewed, including reports and theses, with only 10% contributed by the peer-reviewed scientific literature. Over one-third (36%) of the geographical coordinates were obtained from personal communication, mostly in the form of GPS readings. Geo-positioning was particularly difficult in CSE Asia, where more than a third of the survey sites were geo-positioned using a combination of methods to overcome these difficulties.

Year of Survey

Most of the data assembled derived from surveys conducted from the year 2000 onwards (82%) and from 2005 (66%) (Figure S2.2). Nearly one third of the data (33%) was collected in the years 2007 and 2008, the latter representing the data richest year. The majority of surveys in Africa+ and CSE Asia were conducted in the period between 2005 and 2010 (76% and 66%, respectively).

Age Range of Study Populations

In Table S2.3 the *Pv*PR data are summarised into four groups according to the upper age limit of the study population. The great majority of survey samples (88%) included adults (>20 years of age). This was particularly true for America and CSE Asia, where 97 and 91% of surveys, respectively, sampled this age group. In Africa+ adults were sampled in 70% of surveys.

Diagnostic Methods

Malaria prevalence surveys using only the two most common diagnostic methods, microscopy and rapid diagnostic tests (RDTs), were incorporated into the *Pv*PR database. All surveys from America and Africa+ used microscopy as the examination method. In CSE Asia 2,314 surveys in 2007-2010 used RDTs despite the relative limitation of current tests for diagnosing *P. vivax* infection . Only 399 of those documented used an RDT that targets *P. vivax* specifically (Table S2.4).

Survey Sample Sizes

Sample sizes ranged from one to more than 15,000 individuals per survey. Median sample sizes were 92, 47 and 120 for America, Africa+ and CSE Asia, respectively. Globally, the median sample size was 107 (IQR: 54-246). A total of 112 surveys did not report the number of individuals tested. In these cases, and since the models required a sample size to be recorded, the latter was inferred from additional information provided by the source or assumed to be 50 if no such information was available.

**S2.5 Age-Standardisation**

The *Pv*PR data were reported in a diversity of age ranges. Like *P. falciparum*, population measures of *P. vivax* malaria prevalence are age-dependent . It was therefore necessary to standardise the *Pv*PR survey estimates to a single, representative age group for comparison. All surveys were standardised to the 1 to 99 year age group (*Pv*PR_1-99_) using the same underlying model form implemented previously for *P. falciparum*  based on catalytic conversion models first adapted to malaria by Pull and Grab and described in detail elsewhere .

Sixty-seven studies were used to develop the age-standardization module of the model, which included 1,696 records of disaggregated age data from 67 unique sites. Sixty of the studies were conducted in Indonesia and seven in Papua New Guinea . The number of age strata varied among studies, but ranged from seven to 64 (mean = 25).

**S2.6 Regionalisation**

As in the most recent study to map *P. falciparum* globally , we adopt here a regional modelling strategy whereby separate and independent models are fitted to regional subsets of the global database. The rationale for stratifying the modelling geographically is two-fold. First, the computational resources required to fit the model (i.e. to estimate parameter distributions via Markov chain Monte Carlo (MCMC)) and use the fitted model to generate predictive maps are heavily dependent on the number of data points being considered. The required computational memory (RAM) and processing (CPU time) tend to scale cubically with the number of data. There are also sound statistical reasons for geographic stratification because each regional model is able to fit parameter distributions independently of those in other regions. This has the practical advantage that systematic differences in the spatial heterogeneity of endemicity between regions, or in the relationship between endemicity and environmental covariates can be better represented with regionally bespoke models. In statistical parlance, this feature allows parameter non-stationarity to be captured . Weighed against these advantages is the issue of data availability. Clearly, if a spatial data set is divided into too many spatial regions, or the regions are inappropriately defined, it may mean that some regions have insufficient data with which to fit robust models.

We subdivided the 95 *Pv*MECs globally into four regions, as shown in Figure S2.3. The sizes of the regions were chosen to strike a balance between too little data, which would yield unacceptable levels of uncertainty, and too much data, which would yield unacceptable computational cost. An immediate disadvantage with regional stratifications is the potential for marked discontinuities in predictions along the boundaries when regions are re-joined to make a final global map. Such discontinuities are biologically implausible, as well as being aesthetically unwelcome in presented maps. To mitigate this effect, the stratified data sets were defined so that each region drew information from data both within the region and within a buffer of one decimal degree (approximately 111km at the equator) around the region’s boundary. This had the practical effect of drawing the levels of predicted surfaces from neighbouring regions to within similar ranges around border regions, reducing the potential for discontinuity.

**References**

1. Guerra CA, Hay SI, Lucioparedes LS, Gikandi PW, Tatem AJ, et al. (2007) Assembling a global database of malaria parasite prevalence for the Malaria Atlas Project. Malaria J 6: 17.

2. Gething P, Patil A, Smith D, Guerra C, Elyazar I, et al. (2011) A new world malaria map: *Plasmodium falciparum* endemicity in 2010. Malaria J 10: 378.

3. Mueller I, Galinski MR, Baird JK, Carlton JM, Kochar DK, et al. (2009) Key gaps in the knowledge of *Plasmodium vivax*, a neglected human malaria parasite. Lancet Infect Dis 9: 555-566.

4. Pull JH, Grab B (1974) Simple epidemiological model for evaluating malaria inoculation rate and risk of infection in infants. Bull World Health Organ 51: 507-516.

5. Smith DL, Guerra CA, Snow RW, Hay SI (2007) Standardizing estimates of the *Plasmodium falciparum* parasite rate. Malaria J 6: 131.

6. Elyazar I (2008) Personal communication.

7. Lopansri BK, Anstey NM, Stoddard GJ, Mwaikambo ED, Boutlis CS, et al. (2006) Elevated plasma phenylalanine in severe malaria and implications for pathophysiology of neurological complications. Infect Immun 74: 3355-3359.

8. Baird JK (2008) Personal communication.

9. Syafruddin D, Krisin, Asih P, Sekartuti, Dewi RM, et al. (2009) Seasonal prevalence of malaria in West Sumba district, Indonesia. Malaria J 8: 8.

10. Schultz L, Wapling J, Mueller I, Ntsuke PO, Senn N, et al. (2010) Multilocus haplotypes reveal variable levels of diversity and population structure of *Plasmodium falciparum* in Papua New Guinea, a region of intense perennial transmission. Malaria J 9: 336.

11. Maraga S, Plüss B, Schöpflin S, Sie A, Iga J, et al. (In Press) The epidemiology of malaria in the Papua New Guinea highlands: 7. Southern Highlands Province. PNG Med J.

12. Mueller I, Yala S, Ousari M, Kundi J, Ivivi R, et al. (2007) The epidemiology of malaria in the Papua New Guinea highlands: 6. Simbai and Bundi, Madang Province. PNG Med J 50: 123-133.

13. Mueller I, Ousari M, Yala S, Ivivi R, Sie A, et al. (2006) The epidemiology of malaria in the Papua New Guinea highlands: 4. Enga Province. PNG Med J 49: 115-125.

14. Mueller I, Kundi J, Bjorge S, Namuigi P, Saleu G, et al. (2004) The epidemiology of malaria in the Papua New Guinea highlands: 3. Simbu Province. PNG Med J 47: 159-173.

15. Mueller I, Bjorge S, Poigeno G, Kundi J, Tandrapah T, et al. (2003) The epidemiology of malaria in the Papua New Guinea highlands: 2. Eastern Highlands Province. PNG Med J 46: 166-179.

16. Mueller I, Taime J, Ivivi R, Yala S, Bjorge S, et al. (2003) The epidemiology of malaria in the Papua New Guinea highlands: 1. Western Highlands Province. PNG Med J 46: 16-31.

17. Mueller I, Sie A, Ousari M, Iga J, Yala S, et al. (2007) The epidemiology of malaria in the Papua New Guinea highlands: 5. Aseki, Menyamya and Wau-Bulolo, Morobe Province. PNG Med J 50: 111-122.

18. Mueller I (2008) Personal communication.

19. Mueller I, Widmer S, Michel D, Maraga S, McNamara DT, et al. (2009) High sensitivity detection of *Plasmodium* species reveals positive correlations between infections of different species, shifts in age distribution and reduced local variation in Papua New Guinea. Malaria J 8: 41.

20. Genton B, al-Yaman F, Beck HP, Hii J, Mellor S, et al. (1995) The epidemiology of malaria in the Wosera area, East Sepik Province, Papua New Guinea, in preparation for vaccine trials. I. Malariometric indices and immunity. Ann Trop Med Parasitol 89: 359-376.

21. Kasehagen LJ, Mueller I, McNamara DT, Bockarie MJ, Kiniboro B, et al. (2006) Changing patterns of *Plasmodium* blood-stage infections in the Wosera region of Papua New Guinea monitored by light microscopy and high throughput PCR diagnosis. Am J Trop Med Hyg 75: 588-596.

22. Diggle PJ, Ribeiro PJ (2007) Model-based geostatistics; Bickel P, Diggle P, Fienberg S, Gather U, Olkin I et al., editors. New York: Springer. 228 p.

**Table S2.1.** The inclusion criteria for the MAP *Pv*PR database.

| **Inclusion criterion** | **Original** | **Revised for *Pv*PR database** |
| --- | --- | --- |
| Time of survey | Post 1984 | No change |
| Sample size | ≥50 | >0 |
| Sampling method | Random, community based | No change |
| Intervention studies | Pre-intervention only | No change |
| Spatial duplicate time window | >36 months | >3 months |
| Numerator/denominator | Required | No change |
| Age groups sampled | Children preferred (Africa) | No change |
| Spatial coverage | Points/wide-areas preferred | No change |
| Examination method | Microscopy preferred over RDT | No change |

**Table S2.2.** Exclusions applied to the *Pv*PR database.

|  | **America** | **Africa+** | **CSE Asia** | **Total** |
| --- | --- | --- | --- | --- |
| **Countries with *Pv*PR survey data^†^** | **12** | **19** | **22** | **53** |
| **Total records in starting database** | **408** | **1,643** | **8,143** | **10,194** |
| *Exclusions* |  |  |  |  |
| Large polygons | 5 | 1 | 37 | 43 |
| Small polygons | 7 | 2 | 32 | 41 |
| Surveys with missing month | 8 | 0 | 134 | 142 |
| **Total records for input data set** | **388** | **1,640** | **7,942** | **9,970** |

**^†^**Those countries from which *Pv*PR survey data were available are listed alphabetically by region: America (Bolivia, Brazil, Colombia, Costa Rica, Ecuador, French Guiana, Honduras, Mexico, Nicaragua, Peru, Suriname and Venezuela); Africa+ (Cameroon, Equatorial Guinea, Ethiopia, Ghana, Kenya, Madagascar, Mali, Namibia, Nigeria, Sao Tome and Principe, Saudi Arabia, Senegal, Sierra Leone, Somalia, South Sudan, Sudan, Yemen and Zambia); and CSE Asia (Afghanistan, Bangladesh, Cambodia, China, India, Indonesia, Iraq, Lao People's Democratic Republic, Malaysia, Myanmar, Nepal, Pakistan, Papua New Guinea, Philippines, Solomon Islands, Sri Lanka, Tajikistan, Thailand, Timor-Leste, Turkey, Vanuatu and Viet Nam).

**Table S2.3.** Summary of the most important aspects of the *Pv*PR data by region.

|  | **America** | **Africa+** | **CSE Asia** | **Total** |
| --- | --- | --- | --- | --- |
| **Total records of input data set** | **388** | **1,640** | **7,942** | **9,970** |
| ***Pv*PR values** |  |  |  |  |
| Number of zero records | 171 | 1,288 | 3,631 | 5,090 |
| Mean *Pv*PR | 3.43 | 0.60 | 3.55 | 3.06 |
| Median *Pv*PR | 0.84 | 0.00 | 0.51 | 0.00 |
| Inter-quartile range | 4.01 | 0.00-0.00 | 0.00-3.70 | 0.00-2.97 |
| **Primary source of *Pv*PR data** |  |  |  |  |
| Peer reviewed sources | 214 | 271 | 1,401 | 1,886 |
| Unpublished work | 56 | 386 | 5,760 | 6,202 |
| Reports_†_ | 118 | 983 | 781 | 1,882 |
| **Source of spatial coordinates** |  |  |  |  |
| GPS/Personal communication | 175 | 1001 | 2,459 | 3,635 |
| Encarta | 75 | 103 | 873 | 1,051 |
| GeoNames | - | - | 125 | 125 |
| Google Earth | - | 26 | 81 | 107 |
| Combination | 90 | 110 | 3,472 | 3,672 |
| Other digital gazetteers | 34 | 396 | 299 | 729 |
| Paper source | 13 | 1 | 9 | 23 |
| Map | 1 | 3 | 623 | 627 |
| Not specified | - | - | 1 | 1 |
| **Time period of surveys** |  |  |  |  |
| 1985-1989 | 55 | 86 | 219 | 360 |
| 1990-1994 | 51 | 57 | 479 | 587 |
| 1995-1999 | 120 | 80 | 662 | 862 |
| 2000-2004 | 121 | 164 | 1,291 | 1,576 |
| 2005-2010 | 41 | 1,253 | 5,291 | 6,585 |
| **Upper age sampled** |  |  |  |  |
| <=10 | 2 | 376 | 209 | 587 |
| >10 and <=15 | 7 | 81 | 164 | 252 |
| >15 and <=20 | - | 27 | 294 | 321 |
| >20 | 379 | 1,156 | 7,275 | 8,810 |
| **Diagnostic method** |  |  |  |  |
| Microscopy | 388 | 1,640 | 5,456 | 7,484 |
| RDT | - | - | 2,398 | 2,398 |
| RDT – slide confirmed | - | - | 88 | 88 |
| **Denominator** |  |  |  |  |
| No denominator | 6 | 38 | 68 | 112 |
| 1-49 | 125 | 870 | 1,248 | 2,243 |
| 50-100 | 77 | 292 | 1,985 | 2,354 |
| 101-500 | 149 | 300 | 3,466 | 3,915 |
| >500 | 31 | 140 | 1,175 | 1,346 |
| Median (IQR) | 91.5 (40-198) | 47 (34-108) | 120 (54-281) | 107 (54-246) |

_†_Ministry of Health reports, theses and other unpublished sources.

**Table S2.4.** RDTs used as examination method by surveys in CSE Asia.

| **RDT name** | **Number of records** | **Target species*** |
| --- | --- | --- |
| CareStart Malaria | 28 | ‡ |
| FalciVax | 399 | *Pf*, *Pv* |
| ICT Malaria Pf/Pv | 36 | *Pf*, Pan |
| Not specified | 1,935 | NA |

**Pf* = *P. falciparum*; *Pv* = *P. vivax*, Pan = *Plasmodium* species, NA = not applicable. ^‡^The specific type of CareStart Malaria test was not provided.


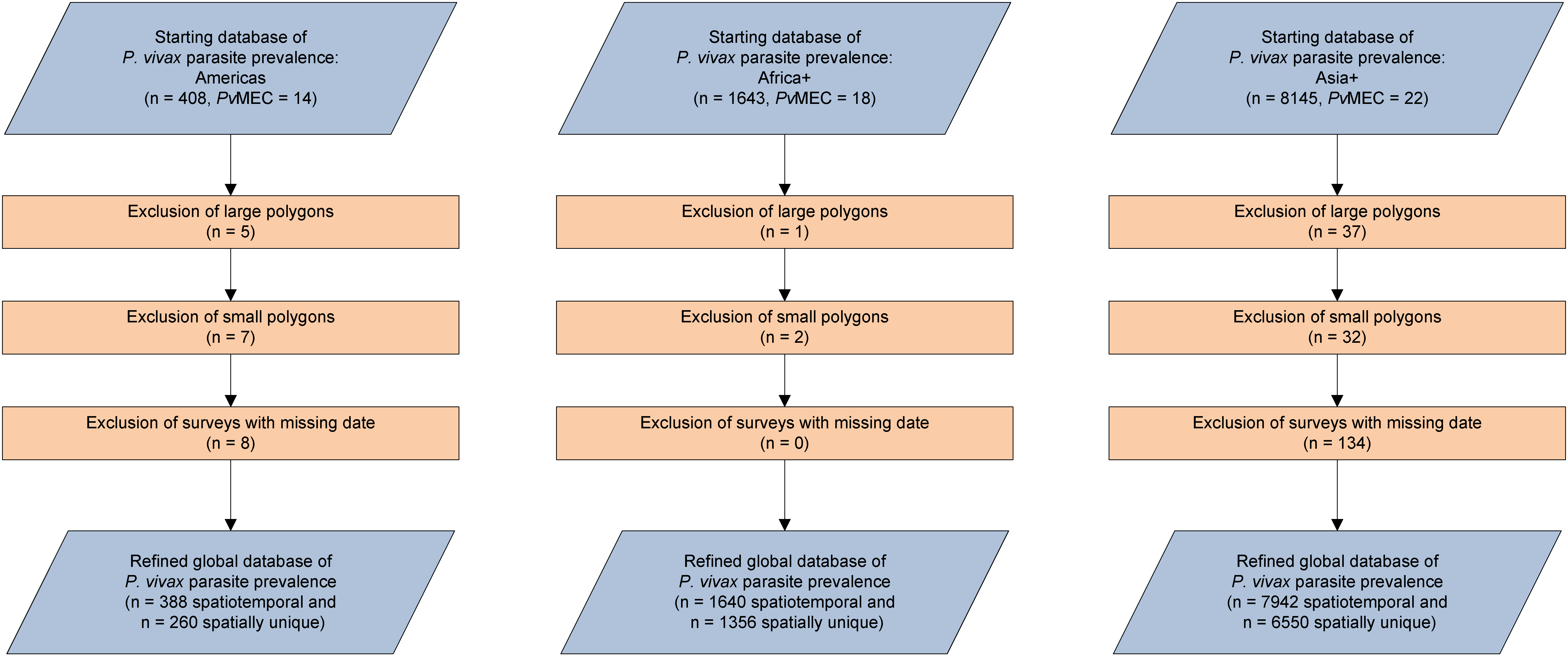


**Figure S2.1**. **Sequence of data exclusion rules for the formulation of a refined global *Pv*PR input data set for modelling.** For each stage of exclusion the number of records excluded are shown in parentheses.

**Figure S2.2.** Cumulative *Pv*PR data record count (y axis) in relation to year of survey (x axis).


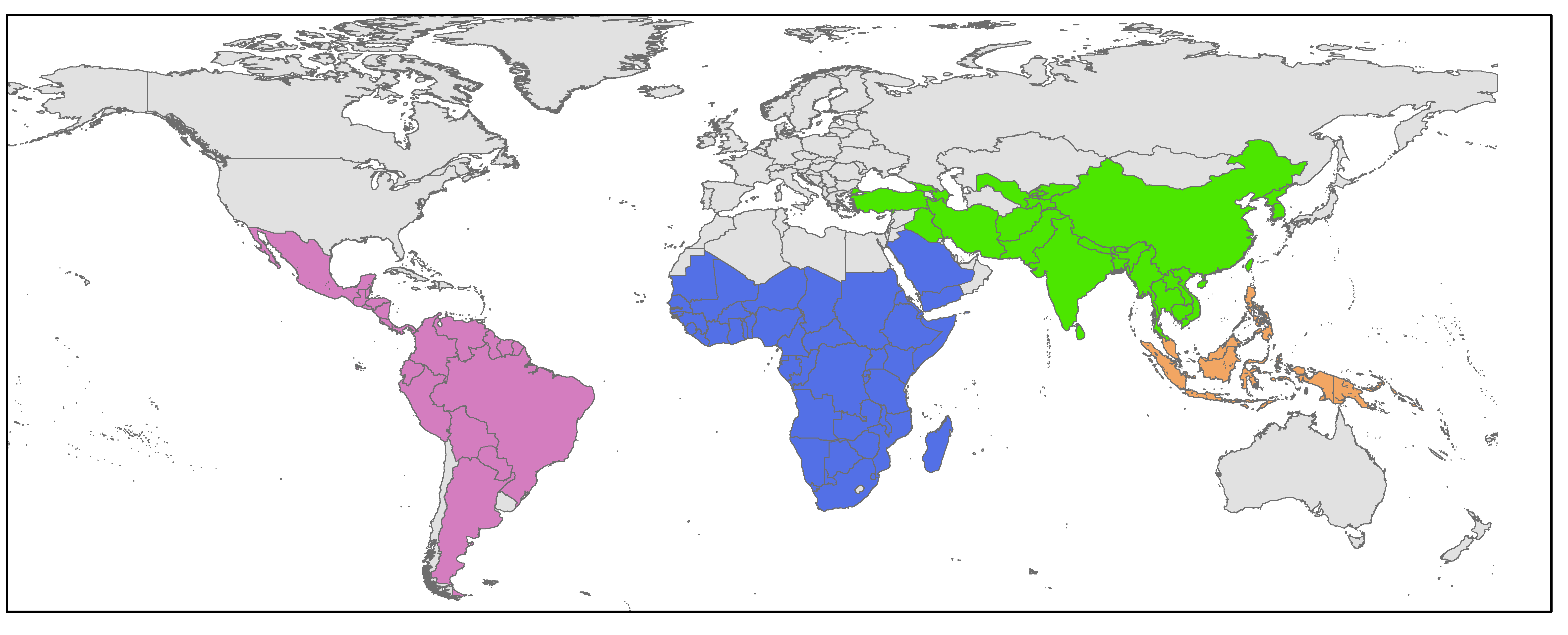


**Figure S2.3. Regional tiles used to stratify modelling.** Pink = Americas; Blue = Africa +; Green = Central Asia; Orange = South East Asia; Grey = Non endemic for *P. vivax*.
