# Supplementary material for: A Long Neglected World Malaria Map: Plasmodium vivax Endemicity in 2010
Source: PLoS Negl Trop Dis. 2012 Sep 6;6(9):e1814. doi: 10.1371/journal.pntd.0001814 (PMC3435256; doi:10.1371/journal.pntd.0001814)
Supplement: Protocol S3 — Bayesian model-based geostatistical framework for predicting Pv PR1–99. S3.1 Bayesian Inference. S3.2 Model Overview. S3.3 Formal Presentation of Model. (DOC) [file pntd.0001814.s003.doc]

**Protocol S3. Bayesian Model-based geostatistical framework for predicting *Pv*PR_1-99_**

**S3.1 Bayesian Inference**

In Bayesian model-based geostatistics, conversion of geo-referenced data to a continuous surface begins with specification of a probability model. Such models are usually collections of interconnected conditional probability statements, for example, “given the number of individuals participating in a sample and the population-wide *Plasmodium vivax* parasite rate (*Pv*PR), the number of positive individuals in that sample is distributed binomially.” These statements, including “prior” probability distributions for basic model parameters, should represent the modeller's understanding of the relationship between the inferential targets (the *Pv*PR surface) and the data (the sample outcomes). The most broadly applicable class of methods for fitting Bayesian probability models is Markov chain Monte Carlo, or MCMC .

**S3.2 Model Overview**

The probability model used in the current study is a modified version of that used in the 2011 iteration of the global *P. falciparum* mapping project . In this supplementary material we describe only the differences between the model used in this paper and that model. The overall approach was to assume that individuals participating in each sample (*Pv*PR survey) were *P. vivax* positive with a probability that was the product of three components: (i) a continuous function of the time and location of the survey, (ii) the proportion of the local population that was Duffy positive, and (iii) a factor that depended on the age range of individuals included in the survey. The continuous function of time and location was modelled as a Gaussian process (or Gaussian random field) . The proportion of the local population that was Duffy positive was obtained from the posterior predictive median map of Duffy negativity obtained by Howes et al. . The distributions of the age-standardisation factors were modelled using a Bayesian version of the procedure described by Smith *et al*. , and inferred based on detailed age-stratified information reported by an updated assembly of surveys.

**S3.3 Formal Presentation of Model**

We present in Figure S3.1 a schematic graphical representation of the full model. Such representations are helpful for visualising complicated probability models. Each of the $N_{i}$individuals in sample $i$ was assumed *P. vivax* positive with probability $\tilde{k}_{i}P'(x_{i},t_{i})(1-d(x_{i},t_{i}))$, where the maximum endemicity $P'(x_{i},t_{i})$was modeled as a transformed Gaussian process; $d(x_{i},t_{i})$ is the proportion of the local population assumed Duffy negative; and the age standardisation factor $\tilde{k}_{i}$, $0\leq\tilde{k}_{i}\leq1$, converted $P'(x_{i},t_{i})(1-d(x_{i},t_{i}))$ to the probability that individuals within the age range reported for study $i$ were *P. vivax* positive, and that the infection was detected, thereby accounting for the influence of age on the probability of detection . The number positive $N_{i}^{+}$was distributed binomially:

$$N_{i}^{+}|N_{i},P'(x_{i},t_{i})\sim^{\text{ind}} \text{Bin}(N_{i},\tilde{k}_{i}P'(x_{i},t_{i})(1-d(x_{i},t_{i}))$$

The Duffy negative proportion of the local population, $d(x_{i},t_{i})$ was assumed equal to the posterior median obtained by Howes et al. . Each age standardisation factor $\tilde{k}_{i}$was assumed drawn independently from a distribution $D_{\tilde{k}}$whose parameters were the lower and upper age limits reported in study $i$, denoted $A_{L,i}$and $A_{U,i}$. The distribution $D_{\tilde{k}}$was obtained using the method described by Gething et al. , applied to 67 age-stratified *P. vivax* parasite rate surveys.

The mapped *Pv*PR surface displays the *P. vivax* parasite rate for all age groups above one year of age (notionally 1-99). Its value at an arbitrary location *x* and time *t* is the product of $P'(x_{i},t_{i})$and another age standardization factor, $k_{1-99}$, which had a probability distribution described by Gething et al. and denoted *D_k_*:

$$\begin{matrix} Pv\text{PR}(x,t)=P'(x,t)(1-d(x,t))k_{1-99}(x,t) \\ k_{1-99}(x,t)\sim^{\text{ind}} D_{k}(1,99) \end{matrix}$$

This composite value gives the probability that an individual selected at random from the population, excluding individuals less than one year old, is *P. vivax* positive.

The coefficient $P'(x,t)$ at arbitrary location $x$and time $t$ was modeled as the inverse logit function applied to a random field $f$evaluated at $(x,t)$, plus an unstructured (random) component :

The components were assumed independent and identically distributed for each location *x* and time *t*, and a relatively diffuse but proper prior was assigned to their variance *V*:

The random field *f* was modeled as a Gaussian process just as in Gething et al. . Both the age standardisation sub-model and the main spatial model were fitted as explained by Gething et al. , using the open-source Bayesian analysis package PyMC .

References

1. Gelman A, Carlin JB, Stern HS (2003) Bayesian data analysis. Texts in Statistical Science. Boca Raton, Florida, U.S.A.: Chapman & Hall / CRC Press LLC. 696 p.

2. Gilks WR, Spiegelhalter DJ (1999) Markov Chain Monte Carlo in practice. Interdisciplinary Statistics. Boca Raton, Florida, U.S.A.: Chapman & Hall / CRC Press LLC.

3. Gething P, Patil A, Smith D, Guerra C, Elyazar I, et al. (2011) A new world malaria map: *Plasmodium falciparum* endemicity in 2010. Malaria J 10: 378.

4. Banerjee S, Carlin BP, Gelfand AE (2004) Hierarchical modeling and analysis for spatial data. Monographs on Statistics and Applied Probability 101. Boca Raton, Florida, U.S.A.: Chapman & Hall / CRC Press LLC.

5. Howes RE, Patil AP, Piel FB, Nyangiri OA, Kabaria CW, et al. (2011) The global distribution of the Duffy blood group. Nat Commun 2: 266.

6. Smith DL, Guerra CA, Snow RW, Hay SI (2007) Standardizing estimates of the *Plasmodium falciparum* parasite rate. Malaria J 6: 131.

7. Patil A, Huard D, Fonnesbeck CJ (2010) PyMC: Bayesian stochastic modelling in Python. J Stat Softw 35: e1000301.


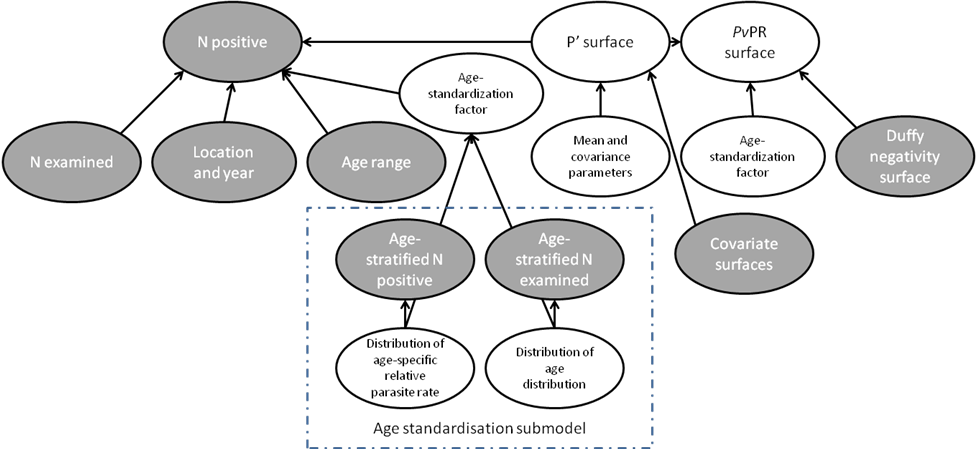


**Figure S3.1 A schematic of the probability model, expressed as a directed acyclic graph.** Ovals represent variables in the model. Grey ovals represent variables that have been observed. Arrows indicate conditional distributions written down in the model. For example, the distribution of the number positive in a parasite rate survey is specified based on the corresponding age-standardisation factor, the underlying maximum *Pv*PR surface *P’* and the time and location of the survey. It is possible to fit the age-standardisation sub-model separately with minimal inconsistency. The maps presented in this study are summaries of the posterior of the upper right-hand node, the *Pv*PR surface.
