# Supplementary material for: A Long Neglected World Malaria Map: Plasmodium vivax Endemicity in 2010
Source: PLoS Negl Trop Dis. 2012 Sep 6;6(9):e1814. doi: 10.1371/journal.pntd.0001814 (PMC3435256; doi:10.1371/journal.pntd.0001814)
Supplement: Protocol S4 — Model validation procedures and additional results. S4.1 Creation of Validation Sets. S4.2 Procedures for Testing Model Performance. S4.3 Validation Results. (DOC) [file pntd.0001814.s004.doc]

**Protocol S4: Model validation procedures and additional results**

Assessing the plausibility of the model output is essential for reliable interpretation of the mapped output. Many different measures of map uncertainty are available. Here, two different aspects of the performance of the predictive model were assessed using a range of validation statistics. This section describes in detail the procedures used to define a validation set, obtain validation data, and compute a series of summary validation statistics and plots, as well as presenting the results of these analyses in full. The procedures follow very closely those used previously for *P. falciparum* , but for completeness are detailed here in full.

**S4.1 Creation of Validation Sets**

Validation statistics obtained *via* prediction of a validation set are representative of model performance only if the validation set itself is a representative sample of the prediction space. Visual examination of the *Pv*PR point data used in this study revealed clear evidence of spatial clustering (Figure 2A, main text). As such, a simple random sample drawn from this set would be similarly clustered and not spatially representative of the predicted *Pv*PR_1-99_ surface as a whole. To generate a spatially representative validation set, the full set of 9,970 data locations was stratified into the four global modelling sub-regions (see Protocol S2, section S2.6) and a spatially declustered sampling procedure was implemented within each. Thiessen polygons were defined around each data location within each region. A Thiessen polygon defines the area closest to each data point in Euclidian space relative to surrounding points. Each datum was then assigned a weight defined as where is the area of the Thiessen polygon surrounding the data location, . A sample of size was drawn without replacement from the regional set where each datum had a probability of selection proportional to its weight, . Those surveys located outside the stable limits of transmission were excluded from selection.

Hold-out sets were thus defined for each modelling sub-region of size = 50, = 175, = 267 and = 530 for the America, Africa+, Central Asia and SE Asia regions, respectively. The model was then re-run in full for each region independently using the corresponding thinned sets of = 338, = 1,465, = 2,398 and = 4,747 data to predict *Pv*PR at the validation locations. In contrast to the main model run in which predictions were made for an annual mean for 2010, the validation run predicted values for the time corresponding to the mid-point of each validation survey to enable fairer comparisons of the observed and predicted *Pv*PR values. We evaluated here the ability of the model to predict *Pv*PR within the age limits reported by each study, rather than age-adjusted *Pv*PR_1-99_. This was deemed a more thorough test of the overall predictive fidelity of the model, because generating predictions for non-standardised age-ranges required an additional age-correction step, and better represented the target quantities generated by the model.

**S4.2 Procedures for Testing Model Performance**

Predictive performance of the model was tested using two different approaches: the ability of the model to (i) predict point-values of *Pv*PR at unsampled locations; and (ii) provide realistic measure of uncertainty for each prediction.

Predicting Point Values of *Pv*PR

The validation procedure generated = 1,022 point estimates of *Pv*PR, where point estimates were calculated using the mean of each predicted posterior distribution. This set of point estimates (where the asterisk denotes a prediction) was then compared to the corresponding set of observed *Pv*PR values at the validation locations. The ability of the model to predict point-values of *Pv*PR at unsampled locations was quantified using three simple summary statistics: the correlation coefficient between the predicted and actual set, the mean prediction error (ME) defined as:

, (S4.1)

and the mean absolute prediction error (MAE) defined as:

(S4.2)

The correlation coefficient provides a straightforward measure of linear association between the data and prediction sets, the ME provides a measure of the bias of the predictor (the overall tendency to over or under predict *Pv*PR values), and the MAE provides a measure of the mean accuracy of individual predictions (the average magnitude of difference between each actual and predicted value). ME and MAE values were presented as both absolute values and as a proportion of the mean *Pv*PR in each region as calculated from the validation set. A scatter plot was also generated as a visualisation of the correspondence between point estimates of *Pv*PR and the corresponding known values.

A sample semi-variogram was calculated from standardised model residuals to assess the presence of residual spatial autocorrelation unexplained by the model output. Standardised Pearson residuals were defined for each validation location as:

(S4.3)

where is the number of individuals surveyed in survey , is the age-standardised number of *P. vivax* positive responses in that survey and is the corresponding point-prediction of *Pv*PR. This standardisation follows established procedures and rescales the raw model residuals to account for their variance characteristics as proportion values. Following the procedure outlined by Diggle and Ribeiro , this sample semi-variogram was compared to a Monte Carlo envelope computed from 99 random permutations of the same residual set. This envelope represents the range of semi-variograms that could be expected by chance in the absence of any spatial structure. Where the semi-variogram of interest lies entirely within this envelope, it can be considered to display no significant spatial structure.

Providing Realistic Measures of Uncertainty for Each Prediction

Posterior distributions arising from Bayesian models provide an estimate of the relative probability of a particular outcome and can be used to characterize uncertainty of prediction . Our model generated a posterior distribution for each unsampled location and a procedure was implemented to test how well the validation set of 2,386 posterior distributions captured the true uncertainty in our model output. A widely used summary measure extracted from predicted posterior distributions is the credible interval (CI), which defines a range of candidate values associated with a specified predicted probability of occurrence. The 95% CIs, for example, are commonly reported around parameter estimates and define the range of possible values for that parameter that has a 0.95 probability of containing the true value. Credible intervals can be extracted from a posterior distribution for any specified level of probability, and can be tested in a validation procedure against the actual proportion of true values falling within different intervals. In a perfect model, for example, 95% of true values should fall within the 95% CI predicted at each location, 50% within the 50% CI, and so on. In this study, we implemented a procedure using this rationale to test the extent to which predicted posterior distributions at each location provided a suitable measure of uncertainty. Working through 100 progressively narrower CIs, from the 99% CI to the 1% CI, each was tested by computing the actual proportion of held-out prevalence observations that fell within the predicted CI. Plotting these actual proportions against each predicted CI level allowed the overall fidelity of the posterior probability distributions predicted at the held-out data locations to be assessed.

**S4.3 Validation results**

Examination of the mean error in the generation of the *P. vivax* malaria endemicity point-estimate surface (Figure S4.1) revealed minimal overall bias in predicted *Pv*PR with a global mean error of -0.46 (Americas -1.38, Africa+ -0.03, Central Asia -0.43, South East Asia -0.43), with values in units of *Pv*PR on a percentage scale (Table S4.1). The global value thus represents an overall tendency to underestimate prevalence by just under half of one percent. The mean absolute error, which measures the average magnitude of prediction errors, was 2.48 (Americas 5.05, Africa+ 0.53, Central Asia 1.52, South East Asia 3.37), again in units of *Pv*PR (Table S4.1). These values give an indication of the consequences of taking values from the point-estimate map as operational estimates of endemicity in each pixel. The estimate is nearly unbiased (i.e. mean errors are small), but variance between predicted and observed endemicities (i.e. mean absolute errors) can be substantial due to the short-range heterogeneity observed in the data and the patchy distribution of the dataset. We have provided elsewhere a more in-depth discussion on approaches to utilising the point-estimate map and associated posterior distribution estimates in downstream quantitative analyses. A semi-variogram of model residuals (Figure S4.1B), defined as Pearson residuals divided by sample size, showed minimal spatial structure. This indicates that the model explains nearly all spatially autocorrelated variation in the observed data, with the unexplained variation being unstructured noise.

The probability-probability plot comparing predicted quantiles with observed coverage fractions (Figure S4.1C) shows the fraction of the observations that were actually contained within each predicted credible interval. This plot illustrates a high degree of fidelity in the predicted quantiles or, put simply, that the predictive distribution is a good representation of the uncertainty in our predictions.

**References**

1. Gething P, Patil A, Smith D, Guerra C, Elyazar I, et al. (2011) A new world malaria map: *Plasmodium falciparum* endemicity in 2010. Malaria J 10: 378.

2. Hay SI, Guerra CA, Gething PW, Patil AP, Tatem AJ, et al. (2009) A world malaria map: *Plasmodium falciparum* endemicity in 2007. PLoS Med 6: e1000048.

3. McCullagh P, Nelder JA (1989) Generalized Linear Models. Boca Raton, Florida: Chapman and Hall / CRC Press. 540 p.

4. Clements ACA, Moyeed R, Brooker S (2006) Bayesian geostatistical prediction of the intensity of infection with *Schistosoma mansoni* in East Africa. Parasitology 133: 711-719.

5. Diggle P, Moyeed R, Rowlingson B, Thomson M (2002) Childhood malaria in The Gambia: a case-study in model-based geostatistics. J Roy Stat Soc C-App 51: 493-506.

6. Diggle PJ, Ribeiro PJ (2007) Model-based geostatistics; Bickel P, Diggle P, Fienberg S, Gather U, Olkin I et al., editors. New York: Springer. 228 p.

7. Congdon P (2003) Applied Bayesian Modelling. Chichester: John Wiley and Sons Ltd. 457 p.

8. Gething PW, Noor AM, Gikandi PW, Hay SI, Nixon MS, et al. (2008) Developing geostatistical space-time models to predict outpatient treatment burdens from incomplete national data. Geogr Anal 40: 167-188.

9. Goovaerts P (2001) Geostatistical modelling of uncertainty in soil science. Geoderma 103: 3-26.

10. Moyeed RA, Papritz A (2002) An empirical comparison of kriging methods for nonlinear spatial point prediction. Math Geol 34: 365-386.

11. Patil AP, Gething PW, Piel FB, Hay SI (2011) Bayesian geostatistics in health cartography: the perspective of malaria. Trends Parasitol 27: 245 - 252.

**Table S4.1. Summary of the validation statistics for predicting continuous *Pv*PR by region.** The mean of each predicted posterior distribution was used as the point estimate of *Pv*PR for comparison with observed values. See text for a full explanation on the derivation of these statistics and interpretation of results. C Asia = Central Asia modelling region; SE Asia = South East Asia modelling region.

| **Validation Measure** | **Americas** | **Africa+** | **C Asia** | **SE Asia** | **World** |
| --- | --- | --- | --- | --- | --- |
| Mean error | -1.38 | 0.03 | -0.43 | -0.43 | -0.41 |
| Mean absolute error | 5.05 | 0.53 | 1.52 | 3.37 | 2.48 |
| Correlation | 0.37 | 0.67 | 0.49 | 0.58 | 0.56 |


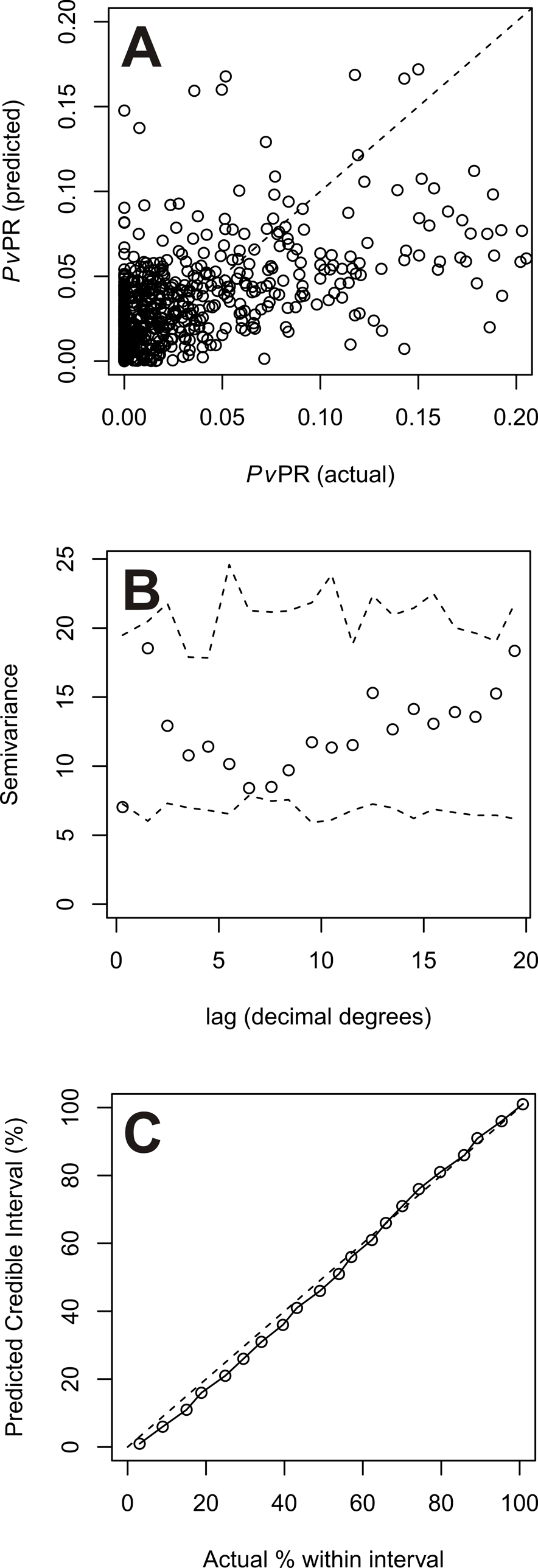


**Figure S4.1.** **Model Validation Plots.** (A) Scatter plot of actual versus predicted point-values of *Pv*PR_1-99_. (B) Sample semi-variogram of standardised model Pearson residuals estimated at discrete lags (circles) and compared to a Monte Carlo envelope (dashed lines) representing the range of values expected by chance in the absence of spatial autocorrelation. (C) Probability-probability plot comparing predicted credible intervals with the actual percentage of true values lying in those intervals. In plots A, and D the 1:1 line is also shown (dashed line) for reference. See text for full explanation of validation procedures and interpretation of results.
